# Impact of Combined mTOR and MEK Inhibition in Uveal Melanoma Is Driven by Tumor Genotype

**DOI:** 10.1371/journal.pone.0040439

**Published:** 2012-07-10

**Authors:** Alan L. Ho, Elgilda Musi, Grazia Ambrosini, Jayasree S. Nair, Shyamprasad Deraje Vasudeva, Elisa de Stanchina, Gary K. Schwartz

**Affiliations:** 1 Laboratory of New Drug Development, Memorial Sloan-Kettering Cancer Center, New York, New York, United States of America; 2 Molecular Pharmacology and Chemistry Program, Memorial Sloan-Kettering Cancer Center, New York, New York, United States of America; 3 Department of Medicine, Weill Medical College of Cornell University, Ithaca, New York, United States of America; The Moffitt Cancer Center & Research Institute, United States of America

## Abstract

Uveal melanomas possess activation of the mitogen-activated protein kinase (MAPK) and phosphoinositide 3-kinase (PI3K)/AKT/mammalian Target of Rapamycin (mTOR) pathways. MAPK activation occurs via somatic mutations in the heterotrimeric G protein subunits *GNAQ* and *GNA11* for over 70% of tumors and less frequently via V600E *BRAF* mutations. In this report, we describe the impact of dual pathway inhibition upon uveal melanoma cell lines with the MEK inhibitor selumetinib (AZD6244/ARRY-142886) and the ATP-competitive mTOR kinase inhibitor AZD8055. While synergistic reductions in cell viability were observed with AZD8055/selumetinib in both *BRAF* and *GNAQ* mutant cell lines, apoptosis was preferentially induced in *BRAF* mutant cells only. *In vitro* apoptosis assay results were predictive of *in vivo* drug efficacy as tumor regressions were observed only in a *BRAF* mutant xenograft model, but not *GNAQ* mutant model. We went on to discover that *GNAQ* promotes relative resistance to AZD8055/selumetinib-induced apoptosis in *GNAQ* mutant cells. For *BRAF* mutant cells, both AKT and 4E-BP1 phosphorylation were modulated by the combination; however, decreasing AKT phosphorylation alone was not sufficient and decreasing 4E-BP1 phosphorylation was not required for apoptosis. Instead, cooperative mTOR complex 2 (mTORC2) and MEK inhibition resulting in downregulation of the pro-survival protein MCL-1 was found to be critical for combination-induced apoptosis. These results suggest that the clinical efficacy of combined MEK and mTOR kinase inhibition will be determined by tumor genotype, and that *BRAF* mutant malignancies will be particularly susceptible to this strategy.

## Introduction

Uveal melanoma is a primary intraocular malignancy that arises from melanocytes within the uveal tract, which includes the iris, ciliary body, and choroid. Over 50% of these patients develop incurable, metastatic disease for which there are no effective therapies [1].

52% to 86% of uveal melanomas possess activation of the mitogen-activated protein kinase (MAPK) pathway [2], [3]. In contrast to cutaneous melanomas, the relevance of *BRAF* (T1799A→V600E) mutations for MAPK activation in uveal melanoma is less clear. Though initial studies of uveal melanoma tumor samples reported that *BRAF* mutations are rare [2]–[6], several groups have detected the mutation in a disproportionate number of uveal melanoma cell lines [3], [7]–[10]. Two studies utilizing more sensitive genetic approaches have reported higher rates of *BRAF* mutation and confirmed that the mutation can be limited to select areas within a tumor [10], [11]. The possibility that techniques to establish cell lines may preferentially select for *BRAF* mutant tumors also can not be excluded.

For over 40% of uveal melanomas, MAPK activation is driven by mutation of *GNAQ* [12], a gene that encodes for the alpha subunit of a heterotrimeric G protein complex (αβγ) that mediates signaling between G-protein–coupled receptors (GPCRs) and downstream effectors. Mutation of codon 209 in the *ras*-like domain blocks intrinsic GTPase activity, keeping Gα in a GTP-bound, constitutively active state. Recently, a mutation at Q209 in the *GNAQ* paralogue gene *GNA11* that also activates MAPK signaling was discovered in over 30% of uveal melanomas [13]. Notably, recent clinical data has demonstrated modest preliminary signals of efficacy in uveal melanoma patients treated with the MEK1/2 inhibitor, selumetinib (AZD6244/ARRY-142886) [14].

It has been hypothesized that activation of the phosphoinositide 3-kinase (PI3K)/AKT/mammalian Target of Rapamycin (mTOR) pathway cooperates with MAPK activation to generate and maintain the malignant phenotype in uveal melanomas. High rates of loss of heterozygosity (LOH) at the *phosphatase and tensin homolog* (*PTEN*) (a negative regulator of the PI3K/AKT/mTOR pathway) locus and mutations within the *PTEN* coding region translates to more than half of uveal melanomas having decreased or complete loss of PTEN expression [15]. Pathway stimulation can also occur via activation of upstream receptor tyrosine kinases (RTKs), including c-kit, insulin-like growth factor type 1 receptor (IGF-1R), and c-met [1].

For inhibition of the PI3K/AKT signaling, effort has been put into targeting mTOR (mammalian Target of Rapamycin), which is a serine/threonine kinase activated by the pathway. The drugs rapamycin and its analogues inhibit mTOR complex 1 (mTORC1), which includes mTOR and other regulatory proteins such as regulatory associated protein of mTOR (Raptor). However, clinical activity with rapamycin and its analogues has been modest to date, and several molecular mechanisms potentially limiting clinical efficacy have been proposed, including: 1) incomplete mTORC1 inhibition, 2) activation of AKT by release of negative feedback pathways regulated by mTORC1 substrates and 3) minimal inhibition of a second rapamycin-resistant complex called mTOR complex 2 (mTORC2), which contains a protein called “rapamycin-insensitive component of mTOR” (Rictor) instead of Raptor. mTORC2 is the protein dependent kinase 2 (PDK2) responsible for phosphorylating AKT at serine 473 [16], which in cooperation with threonine 308 phosphorylation results in full AKT activation. These limitations with mTORC1 inhibitors led to the development of ATP-competitive inhibitors of mTOR which effectively inhibit both mTORC1 and mTORC2 [17]–[19].

In this study, we explored combined MAPK and PI3K/AKT/mTOR pathway inhibition with an ATP-competitive mTOR inhibitor, AZD8055, and the allosteric MEK inhibitor, selumetinib, in uveal melanoma cell lines of various genotypic backgrounds. AZD8055 potently inhibits the mTOR kinase *in vitro* (IC_50_ of 0.8+/−0.2 nM), while exhibiting >1000 fold selectivity against closely related kinases such as PI3K, ATM, and DNA-PK, and no activity against a panel of 260 other kinases at a concentration of 10 µM [17]. Selumetinib is a highly selective, allosteric inhibitor of MEK1/2 that potently inhibits MEK1 *in vitro* with an IC_50_ of 14.1+/−0.79 nM [20]. Utilizing these small molecule inhibitors, we found that the antitumor effectiveness of combined mTOR and MEK inhibition is dependent upon tumor genotype.

## Results

### The AZD8055/selumetinib Combination Synergistically Inhibits BRAF and GNAQ Mutant Tumor Cell Viability

We investigated the impact of dual pathway inhibition with the MEK1/2 inhibitor selumetinib and the mTOR kinase inhibitor AZD8055 upon uveal melanoma cell lines of distinct tumor genotypes. This panel included two cell lines which lack activating mutations in *BRAF*, *GNAQ*, *GNA11*, and *RAS* (Mel290 and C918, referred to as “wild type” or “WT”), two cell lines which possess the V600E *BRAF* mutation (OCM3 and OCM1A, referred to as “BRAF”), and two cell lines which possess activating *GNAQ* mutations at Q209 (92.1 (Q209L) and Mel270 (Q209P), referred to as “GNAQ”); these cell lines were otherwise negative for common mutations in several receptor tyrosine kinases (RTKs), *PIK3CA*, and *AKT1* (**Table S1**). PTEN protein expression was detectable by Western blot in all cell lines (**Figure S8**).

**Figure 1:**
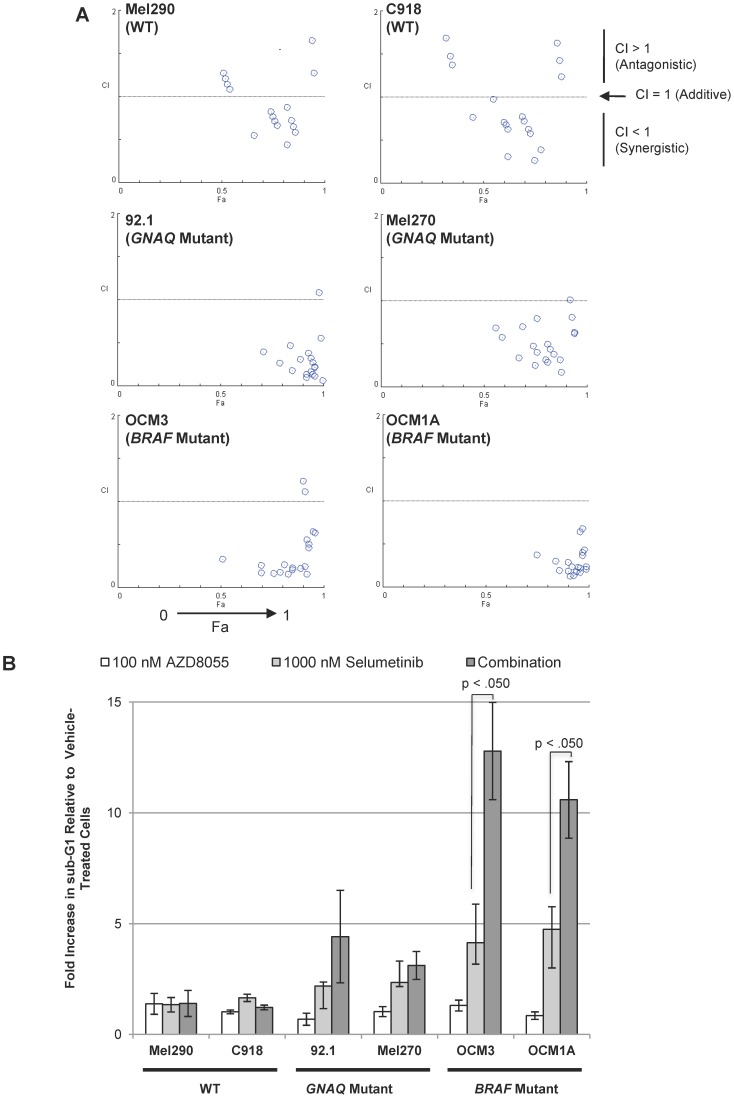
The impact of AZD8055/selumetinib upon uveal tumor cell viability and apoptosis. *A,* AZD8055/selumetinib synergistically inhibited *BRAF* and *GNAQ* mutant tumor cell viability after 96 hours of drug exposure. The graphs are Chou-Talalay plots (*X-axis:* Fa, or fractional activity, reflects the fraction of cellular viability relative to vehicle controls affected by the drug treatment; *Y-axis,* combination index (CI) with <1, >1, and  = 1 indicating synergistic, antagonistic, and additive effects, respectively). Each point represents a different combination of drug concentrations tested. *Concentrations tested:* AZD8055 (0, 20, 50, 100, 1000 nM) and selumetinib (0, 20, 50, 100, 1000, 5000 nM). *B,* sub-G1 fractions were quantified in the uveal melanoma cell line panel following the indicated 48 hour-drug treatments. AZD8055/selumetinib induced apoptosis over either drug alone only in *BRAF* mutant uveal tumor cells, and not in *GNAQ* mutant cells. Results represent the mean of three independent experiments.

The viability of cells treated with various concentrations of AZD8055 (0 to 1000 nM) and selumetinib (0 to 5000 nM) alone and in different combinations were evaluated (50% growth inhibitory values (GI50s) for each compound alone are depicted in **Figure S1**). In both GNAQ and BRAF cells, the AZD8055/selumetinib combination at different dose combinations consistently reduced cell viability by more than 50% (“fractional activity” (Fa) >0.5) in a synergistic manner (combination index (CI) values <1) (**Figure 1A**). The interaction between the two drugs was not effective in WT cells as observed CI values ranged from slightly less than 1 to greater than 1 with lower Fa values than those observed in BRAF and GNAQ cells (**Figure 1A**).

### Selective mTOR and MEK Inhibition with AZD8055/selumetinib Induces Apoptosis Preferentially in BRAF Mutant Uveal Melanoma Cell Lines

We next investigated how differences in cellular viability correlated with apoptosis. 100 nM AZD8055 and 1000 nM selumetinib were utilized for this and all subsequent experiments after it was determined that these are the lowest concentrations that effectively inhibit mTOR and MEK, respectively, in cells (see **Figure S2**).

Results from the apoptosis assays did *not* correlate with the findings from the viability assays. The AZD8055/selumetinib combination increased apoptosis over that observed with either drug alone only in BRAF cells (**Figure 1B**). GNAQ cells demonstrated a modest trend towards increased apoptosis with the combination and no increase in apoptosis was observed in the WT cells (**Figure 1B**). Quantification of apoptosis by Annexin V cell surface staining confirmed these findings (data not shown).

### The AZD8055/selumetinib Combination Induces in vivo Tumor Regression in a BRAF Mutant Xenograft Model, but not in a GNAQ Mutant Model

**Figure 2:**
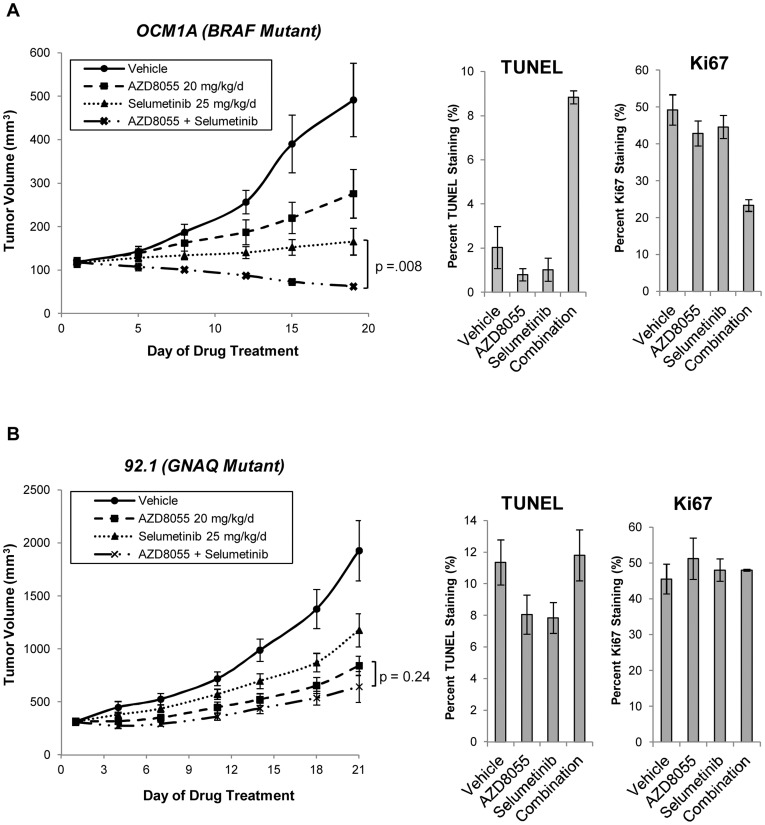
AZD8055/selumetinib induces tumor regression in a *BRAF* mutant, but not *GNAQ* mutant, xenograft model. *A,* AZD8055/selumetinib cooperatively induced tumor regression relative to baseline in a xenograft model with the *BRAF* mutant OCM1A cell line. For detailed methods, please see the *Materials and Methods* section. Briefly, athymic mice were subcutaneously injected with OCM1A cells. Drug treatments began after the tumors were about 100 mm^3^. Animals with established tumors were treated once daily with AZD8055 (20 mg/kg/d) or selumetinib (25 mg/kg/d) alone or in combination for 5 days each week for a total of 3 weeks. Tumors were measured with calipers every 2 to 3 days. Tumor volume was compared between groups of mice at various points in time. Each value represents the mean measurement of 3 to 5 animals. p-value = .008 (Wilcoxon Rank Sum test) for the comparison of selumetinib alone versus the combination **or** AZD8055 alone versus the combination at Day 19. Also, after the fifth drug(s) or vehicle treatment, two animals from each cohort were sacrificed and the tumors were assessed for TUNEL and Ki67 staining. Results in the graphs represent the mean percentages from 4 randomly selected fields; at least 100 cells were counted from each field. *B,* AZD8055/selumetinib failed to induce tumor regressions in the *GNAQ* mutant 92.1 cell line xenograft model. The experiment was conducted as described in *A*. p-value = 0.24 (Wilcoxon Rank Sum test) for the comparison of AZD8055 alone versus the combination. *Error bars, SE*.

We hypothesized that the preferential induction of apoptosis with the combination in *BRAF* mutant cells suggests that combined mTOR and MEK inhibition may be particularly effective for this tumor genotype. To evaluate this *in vivo*, we tested AZD8055 and selumetinib in both a *BRAF* mutant (OCM1A) and *GNAQ* mutant (92.1) xenograft tumor model. In the *BRAF* mutant model, the combination resulted in tumor regressions at Day 19 with tumor volumes that were lower than those achieved with either drug alone (**Figure 2A**, p-value = 0.008 for the comparison of the combination to either AZD8055 or selumetinib alone). Ki67 and TUNEL tumor tissue staining performed after five doses of drug treatment revealed that the combination both decreased tumor proliferation and induced apoptosis (**Figure 2A**; see **Figures S3A** for the immunohistochemistry (IHC) images). In the *GNAQ* mutant model, only a non-statistically significant trend towards enhanced tumor growth inhibition with the combination was achieved; neither tumor regression nor changes in Ki67 and TUNEL staining were observed (**Figure 2B** and **Figure S3B**). Hence, we concluded that the *in vitro* apoptosis assay results were more predictive of *in vivo* drug effectiveness than the *in vitro* viability data, and we subsequently focused our efforts upon investigating the mechanism(s) by which these drugs elicit distinct apoptotic outcomes in the *BRAF* and *GNAQ* mutant uveal tumor genotypes.

### GNAQ Mediates Relative Resistance to AZD8055/selumetinib-induced Apoptosis in GNAQ Mutant Cells

**Figure 3:**
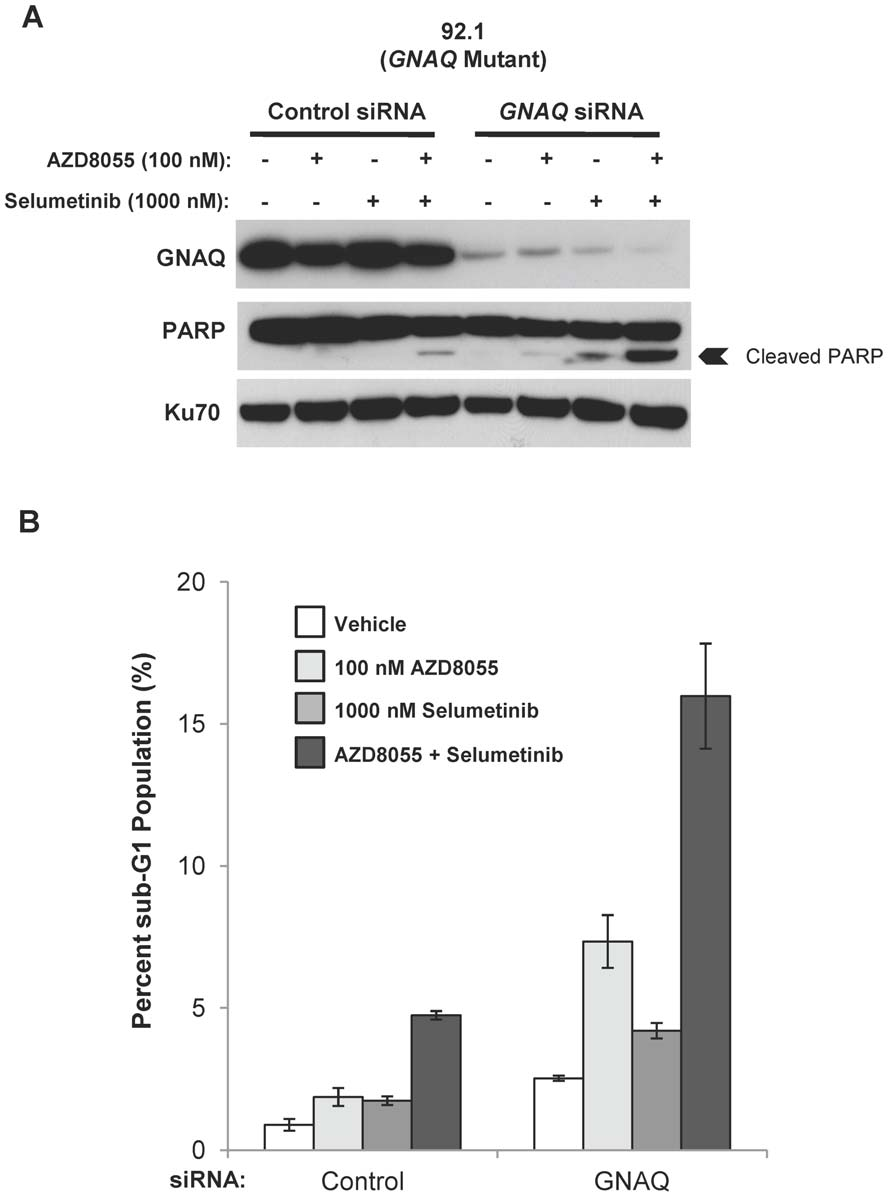
Suppression of GNAQ expression in *GNAQ* mutant cells augments AZD8055/selumetinib-induced apoptosis. *A,* suppression of *GNAQ* expression in the *GNAQ* mutant cell line 92.1 with pooled siRNA constructs resulted in increased PARP cleavage with the AZD8055/selumetinib combination. 92.1 cells were transfected with pooled *GNAQ* targeting siRNA constructs or unrelated control constructs for 24 hours and then treated with drugs for 24 hours (vehicle (denoted by “-”), 100 nM AZD8055, 1000 nM selumetinib, or the combination). Cell lysates were created and Western blots were then performed. The nuclear protein Ku70 was used as a loading control. *B,* suppression of *GNAQ* expression increased the sub-G1 population induced by the AZD8055/selumetinib combination in 92.1 cells. Cells were treated as detailed in *A* and then analyzed by flow cytometry for DNA content. The percentage of sub-G1 cells was quantified. Results are the mean of two independent experiments. *Error bars, SE.*

Given the modest level of apoptosis observed in GNAQ cells, we hypothesized that GNAQ activation may mediate relative resistance to apoptosis induced by combined mTOR and MEK inhibition. In order to determine if GNAQ is necessary to block AZD8055/selumetinib-induced apoptosis, *GNAQ* expression was suppressed with pooled small interfering RNA (siRNA) constructs in the *GNAQ* mutant cell line 92.1 (**Figure 3A**). While suppression of GNAQ expression alone only modestly increased apoptosis, the combination induced higher levels of poly-ADP ribose polymerase (PARP) cleavage (**Figure 3A**) and substantially increased the sub-G1 fraction (**Figure 3B**) in GNAQ siRNA-transfected cells compared to control siRNA-transfected cells (sub-G1 percentages of 16.0% versus 4.7%, respectively). Hence, in these cells GNAQ is essential for countering apoptosis induced by combined MEK and mTOR inhibition.

### Persistent AKT Phosphorylation in AZD8055-treated BRAF Mutant Cells is Mediated by IGF-1R Activation in a MEK-dependent Manner

**Figure 4:**
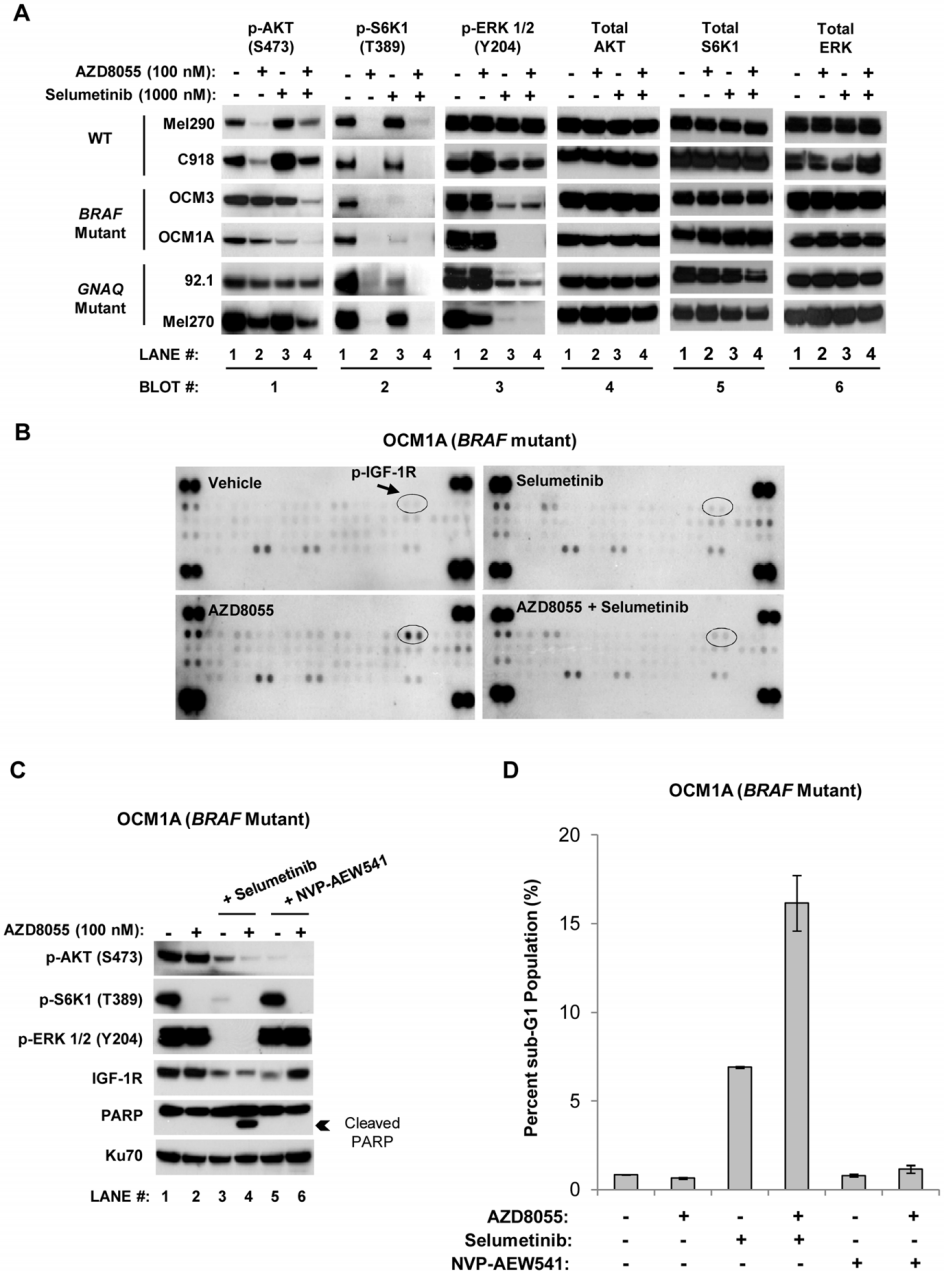
Selumetinib suppresses AKT phosphorylation in AZD8055-treated *BRAF* mutant cells. *A,* uveal melanoma cell lines produced distinct biochemical responses to AZD8055 and selumetinib exposure. Cells were treated with the indicated drugs or vehicle (denoted by “-”) for 24 hours and Western blots were then performed. Total AKT was used as a loading control. Of note, selumetinib alone inhibited S6K1 phosphorylation in BRAF cells and to a lesser extent in GNAQ cells (**lane 1 versus 3 in Blot #2**). Given how effectively AZD8055 inhibited S6K1 phosphorylation, though, it is unlikely that this selumetinib effect significantly contributed to the impact of the combination in BRAF cells. *B,* IGF-1R phosphorylation increased with AZD8055 treatment and was suppressed by selumetinib in the *BRAF* mutant cell line OCM1A. OCM1A cells were treated as described in *A,* and cellular lysates were created and analyzed with phosphorylated receptor tyrosine kinase (RTK) antibody array blots. The blots reflect the phosphorylation status of 42 RTKs. Duplicate spots in the corners of each blot are positive controls. IGF-1R duplicate spots are circled. *C,* inhibition of IGF-1R blocked AKT phosphorylation in OCM1A cells, but did not induce PARP cleavage in combination with AZD8055. Cells were treated with the same drugs and concentrations as detailed in *A* in addition to the IGF-1R small molecule inhibitor NVP-AEW541 at 1000 nM. Cells were treated for 24 hours before Western blots were performed. Ku70 was used as a loading control. *D,* IGF-1R inhibition with NVP-AEW541 failed to induce apoptosis in combination with selumetinib in OCM1A cells. Cells were treated with drugs for 48 hours and analyzed by flow cytometry for DNA content. The percentages of sub-G1 cells were quantified. Results are the mean of two independent experiments. *Error bars, SE.*

To investigate how AZD8055/selumetinib preferentially elicits apoptosis in *BRAF* mutant cells, we assessed the impact of the combination upon the phosphorylation status of mTOR and MEK substrates. In WT cells, AZD8055 inhibited phosphorylation of both the mTORC1 substrate S6 Kinase 1 (S6K1) and the mTORC2 substrate AKT (at serine 473; **Figure 4A** **, lane 2 of Blots #1 and 2**); selumetinib, however, failed to inhibit ERK phosphorylation in a sustained manner (**Figure 4A** **, lane 3 of Blot #3**) due to rebound ERK phosphorylation (see **Figure S2D** and the accompanying figure legend for the selumetinib time course experiment demonstrating this phenomenon). This lack of sustained target inhibition is one possible explanation for the lack of cooperative antitumor effect observed with the combination in these cells. Additionally, AKT phosphorylation was slightly increased with selumetinib in only these cells (**Figure 4A** **, lane 3 of Blot #1**).

In BRAF and GNAQ cells, AZD8055 potently suppressed S6K1 phosphorylation, but failed to completely inhibit AKT phosphorylation (**Figure 4A** **, lane 2 of Blots #1 and #2; Figure S2A**). This was *not* due to ineffective mTORC2 inhibition, but instead rebound AKT phosphorylation: while 100 nM AZD8055 inhibited AKT phosphorylation after 2 hours in all three tumor genotypes, by 24 hours AKT phosphorylation rebounded only in BRAF and GNAQ cells (**Figure S2C**). Interestingly, the addition of selumetinib to AZD8055 substantially decreased AKT phosphorylation in BRAF cells, but not significantly in GNAQ cells (**Figure 4A** **, lane 4 of Blot #1**).

Given evidence in other systems that the release of negative feedback pathways resulting in AKT phosphorylation represents a critical mechanism of intrinsic resistance to mTORC1 inhibition [21], we investigated the mechanism by which AKT is modulated by AZD8055/selumetinib. Utilizing an RTK array assay, we found that mTORC1/mTORC2 inhibition with AZD8055 in the *BRAF* mutant cell line OCM1A increased phosphorylation of the insulin-like growth factor type 1 receptor (IGF-1R) (**Figure 4B**). The addition of selumetinib to AZD8055 decreased the IGF-1R phosphorylation signal (**Figure 4B**).

These results suggest that inhibition of IGF-1R activity may be one mechanism downstream of MEK/ERK inhibition by which selumetinib inhibits AZD8055-induced, rebound AKT phosphorylation. To test this hypothesis, we utilized the small molecule inhibitor NVP-AEW541 [22] to directly inhibit IGF-1R in OCM1A cells. At 1000 nM, NVP-AEW541 inhibited IGF-1R phosphorylation induced by serum or IGF-1 ligand alone (**Figure S4**). In combination with AZD8055, NVP-AEW541 suppressed AKT re-phosphorylation without impacting ERK phosphorylation (**Figure 4C** **, lane 6**), consistent with the model that IGF-1R activation regulates AKT downstream of MEK/ERK activity in these cells. However, despite modulating AKT phosphorylation, the addition of NVP-AEW541 to AZD8055 was *insufficient* to induce apoptosis as assayed by PARP cleavage and quantifying sub-G1 cell populations (**Figure 4C** **, lane 6** and **Figure 4D** **,** respectively). Hence, while selumetinib suppresses AZD8055-mediated activation of the IGF-1R/AKT axis, modulation of these targets alone is not sufficient for the apoptosis induced by the AZZD8055/selumetinib combination, implying that other target(s) downstream of selumetinib-mediated ERK inhibition is (are) critical for the induction of apoptosis.

### AZD8055/selumetinib Inhibition of 4E-BP1 Phosphorylation in BRAF Mutant Cells also does not Contribute to Apoptosis

4E-BP1 (eIF4E binding protein-1) has recently been implicated as the key protein target cooperatively modulated by an AKT and MEK inhibitor combination to induce apoptosis in tumors harboring both *RAS* and *PIK3CA* mutations [23]. Therefore, we investigated the potential role of 4E-BP1 in AZD8055/selumetinib-induced apoptosis for *BRAF* mutant uveal melanomas.

4E-BP1 is an mTORC1 substrate that negatively regulates translation by binding and blocking the function of eIF4E, an initiation factor that binds to the 5′ mRNA cap structure to promote mRNA translation. mTORC1 activity leads to 4E-BP1 phosphorylation at several sites (T37/T46, S65 and T70 [24]), which results in 4E-BP1 dissociation from eIF4E and activation of cap-dependent translation [25].

**Figure 5:**
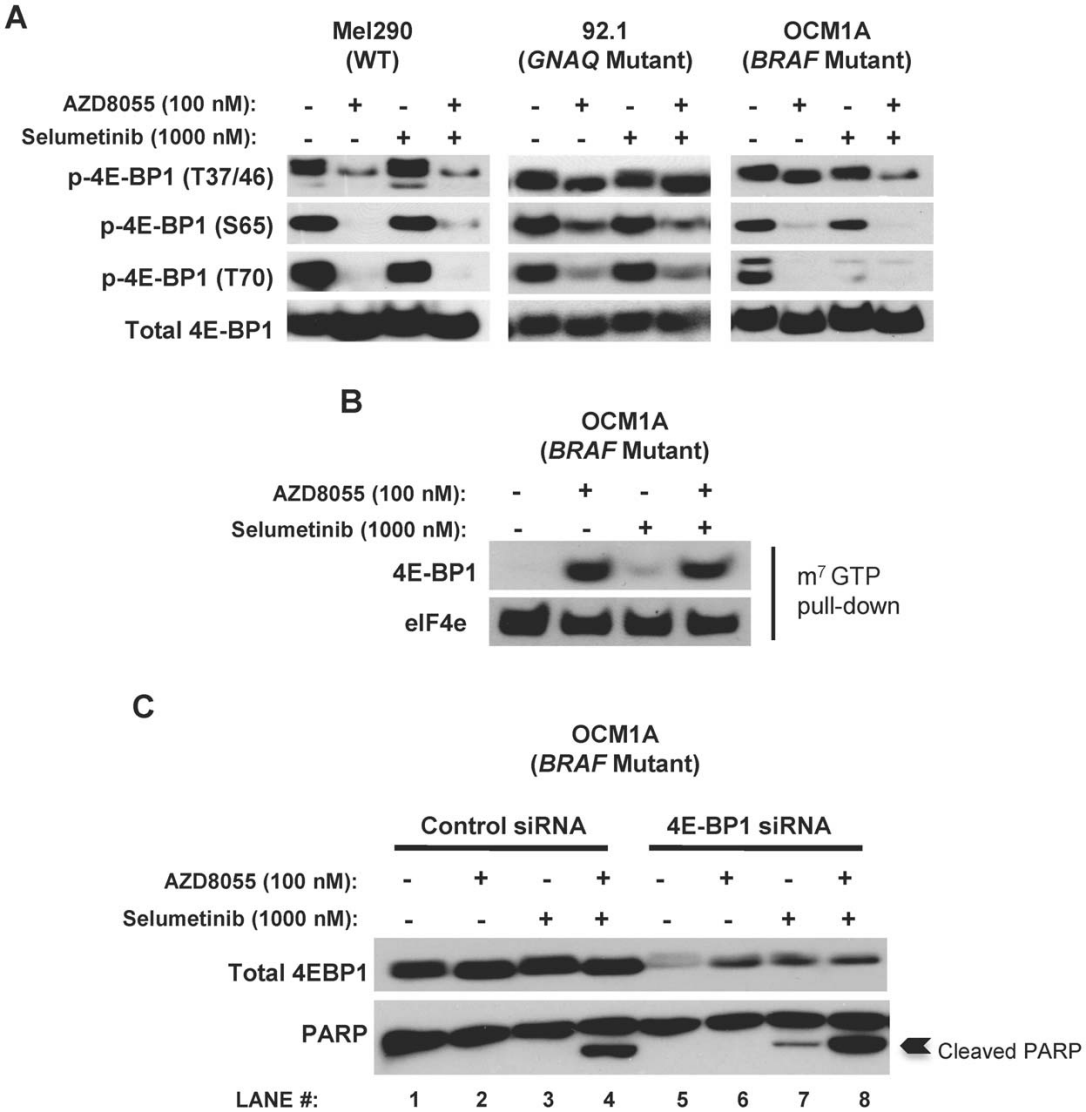
4E-BP1 phosphorylation is modulated by AZD8055/selumetinib, but does not regulate *BRAF* mutant cell survival. *A,* the AZD8055/selumetinib combination cooperatively suppressed 4E-BP1 phosphorylation at T37/46 in the *BRAF* mutant cell line OCM1A. Cells were treated with the indicated drugs for 24 hours. Western blots were then performed. Total 4E-BP1 was used as a loading control. *B,* the capability of 4E-BP1 binding to the mRNA cap complex was not impacted by the AZD8055/selumetinib combination in OCM1A cells. A cap binding assay was performed (referenced in the *Materials and Methods* section). Briefly, cells were treated with the indicated drugs for 24 hours. Cell lysates were created and then incubated with m^7^ GTP sepharose beads to capture all 4E-BP1 and eIF4E proteins that are capable of binding to an mRNA cap complex. The bead-associated proteins were analyzed by Western blot. *C,* suppression of 4E-BP1 expression did not diminish apoptosis as assessed by PARP cleavage in OCM1A cells. Cells were transfected with siRNA constructs targeting 4E-BP1 or unrelated control constructs for 48 hours. Cells were then treated with the indicated drugs for 24 hours. Western blots were performed.

Phosphorylation at all the 4E-BP1 sites assayed was inhibited with AZD8055 alone in WT cells. In the *GNAQ* mutant cell line 92.1 and *BRAF* mutant cell line OCM1A, 4E-BP1 phosphorylation at T37/46 was only modestly suppressed by AZD8055; phosphorylation at S65 and T70 was more effectively inhibited by AZD8055 in these cells (**Figure 5A**). The addition of selumetinib further suppressed 4E-BP1 phosphorylation at T37/46 in only BRAF cells, not GNAQ cells (**Figure 5A**). However, an mRNA cap binding assay demonstrated that this further reduction in T37/46 phosphorylation did not increase the capacity for 4E-BP1 to associate with m^7^ GTP Sepharose beads (which mimics the 5′ cap of untranslated mRNAs) in the BRAF cells, suggesting no significant change upon 4E-BP1 function (**Figure 5B**). Furthermore, pooled siRNA construct-mediated downregulation of 4E-BP1 (which reportedly protected tumors cells from apoptosis induced by combined AKT and MEK inhibition [23]) failed to reduce PARP cleavage induced by the combination, confirming that 4E-BP1 is not an essential regulator of apoptosis in *BRAF* mutant cells (**Figure 5C** **, lane 4 versus lane 8**).

### Combined mTORC2 Suppression and MEK Inhibition with Selumetinib is Sufficient to Induce Apoptosis in BRAF Mutant Cells

**Figure 6:**
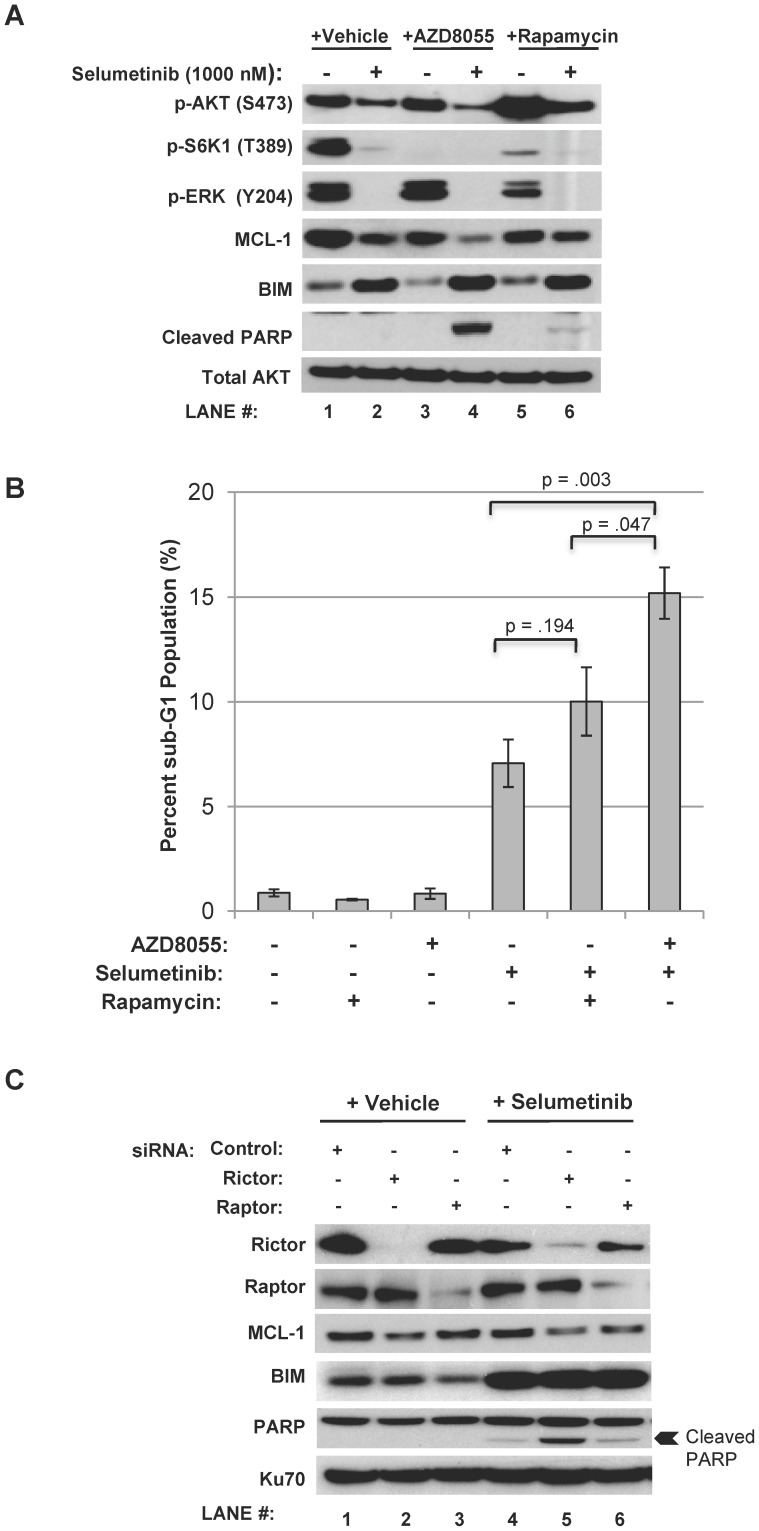
mTORC2 inhibition in combination with selumetinib induces apoptosis in *BRAF* mutant cells. *A,* mTORC1 inhibitor rapamycin in combination with selumetinib failed to induce apoptosis as evidenced by a lack of PARP cleavage in the *BRAF* mutant cell line OCM1A. Cells were treated with the indicated drugs (vehicle, 100 nM AZD8055, 1000 nM selumetinib, and 10 nM rapamycin) for 24 hours. Western blots were then performed. Total AKT was used as a loading control. *B,* rapamycin in combination with selumetinib failed to substantially increase the sub-G1 fraction in the OCM1A cell line. Cells were treated as detailed in *A* and then analyzed by flow cytometry for DNA content; the sub-G1 fraction was quantified. Results are the mean of four independent experiments. *Error bars, SE*. *C,* suppression of Rictor and mTORC2 activity led to MCL-1 downregulation and PARP cleavage in combination with selumetinib in the OCM1A cell line. Cells were transfected with pooled siRNA constructs targeting Rictor or Raptor or an unrelated control construct for 48 hours. Cells were then treated with either vehicle or 1000 nM selumetinib for 24 hours and a Western blot was then performed. Ku70 was used as a loading control.

We next compared the impact upon mutant *BRAF* cell apoptosis of mTORC1 versus mTORC2 inhibition in combination with selumetinib MEK inhibition. First, AZD8055 effects were compared to those of rapamycin, an mTORC1 inhibitor. As predicted, rapamycin suppressed mTORC1 substrate phosphorylation and increased AKT phosphorylation (presumably via release of negative feedback regulation), which was incompletely reduced by selumetinib (**Figure 6A**). Less apoptosis (**Figure 6B**) and PARP cleavage (**Figure 6A** **, lane 4 versus 6**)) was observed with the rapamycin/selumetinib combination compared to the AZD8055/selumetinib combination. Hence, mTORC1 inhibition with rapamycin alone is not sufficient to replicate the apoptosis produced with AZD8055.

mTOR has been shown to stimulate cell survival by promoting expression of myeloid leukemia sequence-1 (MCL-1), a pro-survival member of the bcl-2 family of genes [26]. In epidermal growth factor receptor (EGFR) mutant non-small cell lung cancer cells, MCL-1 downregulation by a PI3K/mTOR kinase inhibitor in combination with BIM upregulation (a pro-apoptotic member of the bcl-2 family) by a MEK inhibitor has been shown to be critical for the induction of apoptosis [27]. We investigated whether or not differences in the regulation of MCL-1 and BIM levels correlated with the differences in apoptosis observed in this study. In all contexts, BIM was upregulated with selumetinib (**Figure 6A** **, lanes 2, 4, and 6**), consistent with previously published data [28], [29]. While all the drugs alone reduced MCL-1 levels to some degree, the AZD8055/selumetinib combination cooperatively reduced MCL-1 to the lowest levels observed with any condition (**Figure 6A** **, lane 4**); the rapamycin/selumetinib combination failed to further decrease MCL-1 levels over that observed with either drug alone (**Figure 6A** **, lane 4 versus 6**).

Taking a previously published approach [30], we next utilized pooled siRNA constructs targeting Raptor and Rictor to evaluate whether the inhibition of mTORC1 or mTORC2 in isolation, respectively, is sufficient to recapitulate the MCL-1 and apoptosis effects observed with AZD8055. Partial Rictor downregulation was achieved with a concomitant decrease in MCL-1 levels compared to control siRNA-transfected cells (**Figure 6C** **, lane 2 versus 1**). Alternatively, partial suppression of Raptor expression failed to reduce MCL-1 levels relative to control siRNA-transfected cells (**Figure 6C** **, lane 1 versus 3**). With the addition of selumetinib, cells with suppressed Rictor levels achieved modestly lower MCL-1 levels and BIM upregulation, resulting in an induction of apoptosis as indicated by the appearance of cleaved PARP that was not observed with raptor siRNA (**Figure 6C** **, lane 5**). These observations suggest that in *BRAF* mutant uveal melanoma the modulation of MCL-1 levels and apoptosis by AZD8055 in combination with selumetinib may be dependent upon mTORC2, not mTORC1, inhibition.

### AZD8055/selumetinib-induced Apoptosis in BRAF Mutant Cells is Dependent Upon Reducing MCL-1 Levels

**Figure 7:**
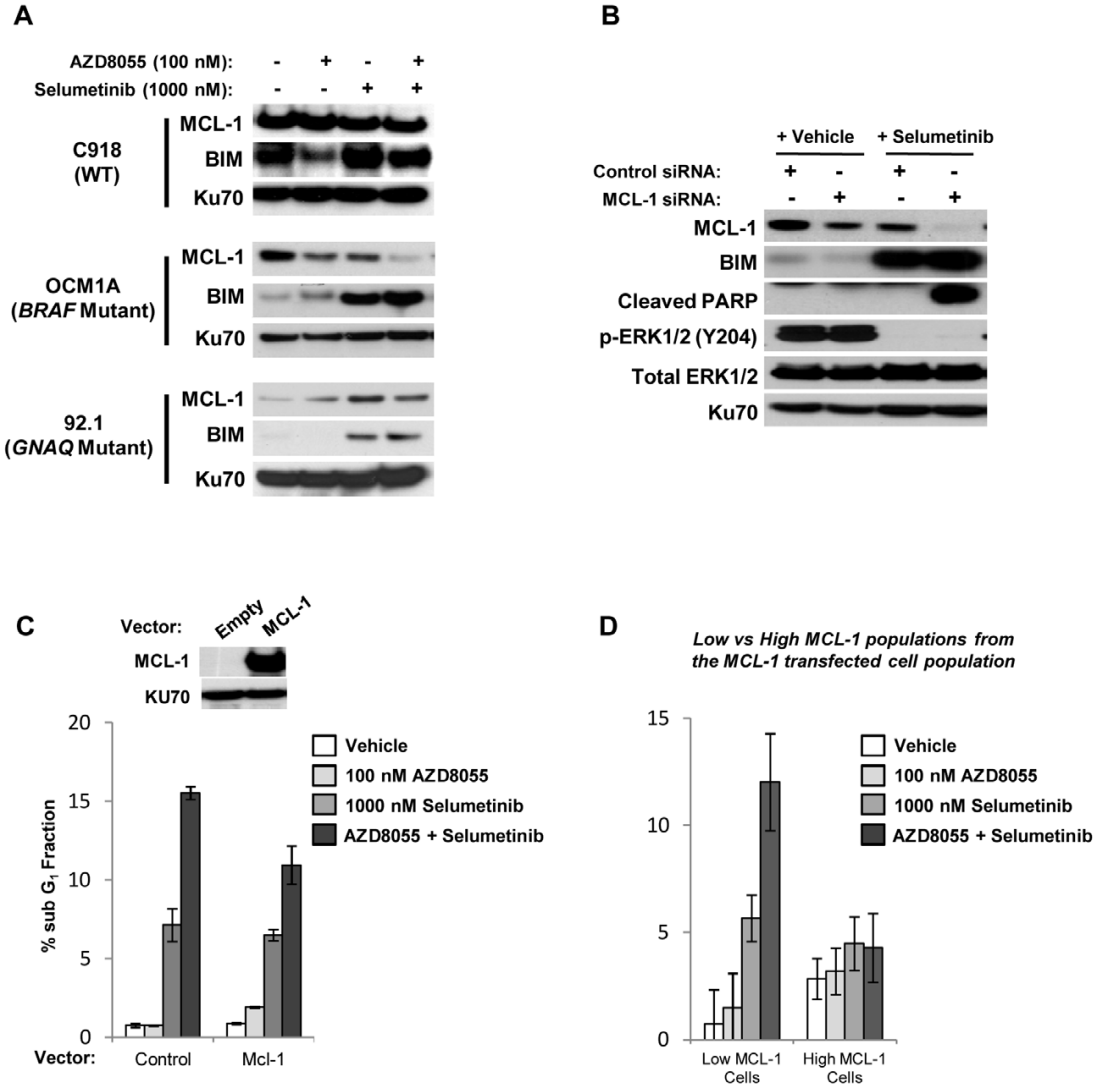
Modulation of MCL-1 by AZD8055/selumetinib contributes to apoptosis in *BRAF* mutant cells. *A,* the AZD8055/selumetinib combination cooperatively suppressed MCL-1 and induced BIM in the *BRAF* mutant OCM1A cell line. Cells were treated with the indicated drugs (vehicle denoted by “-”) for 24 hours and Western blots were then performed. Ku70 was used as a loading control. *B,* suppression of MCL-1 levels via targeting siRNA constructs in combination with selumetinib treatment was sufficient to induce apoptosis as evidenced by increased PARP cleavage in the OCM1A cell line. Cells were transfected with pooled siRNA constructs targeting MCL-1 or unrelated control constructs for 48 hours and then treated with 1000 nM selumetinib for 24 hours. Western blots were then performed. Ku-70 was used as a loading control. *C,* MCL-1 overexpression in the OCM1A cell line reduced AZD8055/selumetinib induced apoptosis. Cells were transiently transfected with an *MCL-1* cDNA expression plasmid under the control of a constitutively active viral promoter or an empty vector for 48 hours. Western blot was performed to confirm overexpression of MCL-1. Cells were then treated with the indicated drugs for 24 hours. Cells were analyzed by flow cytometry for DNA content; sub-G1 populations were quantified. *D* bi-parametric flow cytometry demonstrated that apoptosis was preferentially induced by AZD8055/selumetinib in low MCL-1 expressing cell populations. Cells were transiently transfected with MCL-1 as detailed in *C*. The low- and high- MCL-1 expressing cell populations from these MCL-1 transfected cells were then detected and analyzed by bi-parametric flow cytometry for DNA content in addition to MCL-1 expression level (please see **Figure S6** for the flow cytometry plots). Depicted results are representative of two independent experiments. *Error bars, SE*.

We went on to examine the levels of MCL-1 and BIM expressed in several uveal melanoma cell lines following exposure to AZD8055 and selumetinib (**Figure 7A**). While selumetinib upregulated BIM levels in WT, BRAF, and GNAQ cells, only in the *BRAF* mutant OCM1A cell line was the level of MCL-1 decreased by AZD8055 and selumetinib cooperatively (**Figure 7A**).

To explore if MCL-1 downregulation in BRAF cells is sufficient to induce apoptosis in combination with selumetinib, MCL-1 levels in OCM1A cells were reduced with pooled siRNA constructs. Transfection of MCL-1 targeting siRNAs reduced MCL-1 expression (relative to an unrelated control siRNA) to a level comparable to that observed with AZD8055 alone (**Figure 7B**). While selumetinib in combination with the control siRNA downregulated MCL-1 and increased BIM levels, it was only with further MCL-1 suppression achieved with the targeting siRNAs that the addition of selumetinib induced apoptosis as detected by increased PARP cleavage (**Figure 7B**).

We next transfected into OCM1A cells a plasmid driving MCL-1 expression under the control of a constitutively active viral promoter in order to determine if MCL-1 downregulation is necessary for AZD8055/selumetinib-induced apoptosis (**Figure 7C**). Overexpression of MCL-1 rescued cells from apoptosis induced by the combination, decreasing the apoptosis fraction of OCM1A cells from 15.5% in vector-transfected cells to 10.9% in MCL-1-transfected cells (**Figure 7C**). We also analyzed MCL-1 transfected cells by bi-parametric flow cytometry for both DNA content and MCL-1 levels to separate out low- and high-expressing MCL-1 cells and then compare the differences in sub-G1 percentage between these two subsets (**Figure 7D** and **Figure S5**). This analysis revealed that apoptosis was induced with the combination only in the subset of cells expressing lower levels of MCL-1, while the drug combination failed to induce more apoptosis than that observed with selumetinib alone in the high MCL-1 expressers (**Figure 7D** and **Figure S5**). In the xenograft model of the *BRAF* mutant cell line OCM1A, Western analysis of frozen tumor tissue revealed that cooperative MCL-1 downregulation and increased BIM levels were also achieved *in vivo* (**Figure S6**). Taken together, these studies demonstrate that MCL-1 downregulation is both necessary and sufficient to induce apoptosis with selumetinib, and strongly argues that MCL-1 is a singular target that is cooperatively modulated by the combination to induce apoptosis in *BRAF* mutant uveal melanoma.

## Discussion

Concomitant activation of the MAPK and PI3K/AKT pathways in tumor cells confers relative resistance to drugs targeting either pathway in isolation. AKT activation in *BRAF* mutant cutaneous melanomas mediates resistance to MEK inhibition with selumetinib [31]. *BRAF* and *RAS* mutations mediate resistance to AKT targeted agents [23]. Alternatively, drug combinations that inhibit both pathways may be more clinically effective for tumors with evidence of dual pathway activation, including those with RTK mutation/activation [27], [32], other genetic alterations of the pathway (*RAS, PIK3CA, BRAF* mutations and/or *PTEN* loss) [23], [28], [33]–[37], or simply expression of phosphorylated AKT or ERK [31], [38].

In this study, we found that uveal cancer cell fate in response to combined MEK and mTOR inhibition was closely correlated to tumor genotype. AZD8055/selumetinib did not confer cooperative antitumor effects in WT cells, possibly related to incomplete suppression of MAPK activity with selumetinib. Alternatively, the combination did synergistically suppress the viability of both *BRAF* and *GNAQ* mutant cells; however, apoptosis was only induced in *BRAF* mutant cells. Ultimately, *in vitro* measurement of apoptosis correlated better than cell viability to *in vivo* effectiveness as tumor regressions were observed only in the *BRAF* mutant xenograft model. These distinct drug induced fates may in fact reflect biologic differences in how *BRAF* and *GNAQ* mutations activate MAPK signaling: while the former directly activates MEK→ERK activity, the latter does so via protein kinase C (PKC) activation which can mediate cell survival signals via several pathways parallel to MAPK [1]. The observation that suppression of GNAQ expression in *GNAQ* mutant cells predisposes to AZD8055/selumetinib-induced apoptosis argues that GNAQ activity in these cells activates MEK- and mTOR- independent pro-survival signals.

**Figure 8:**
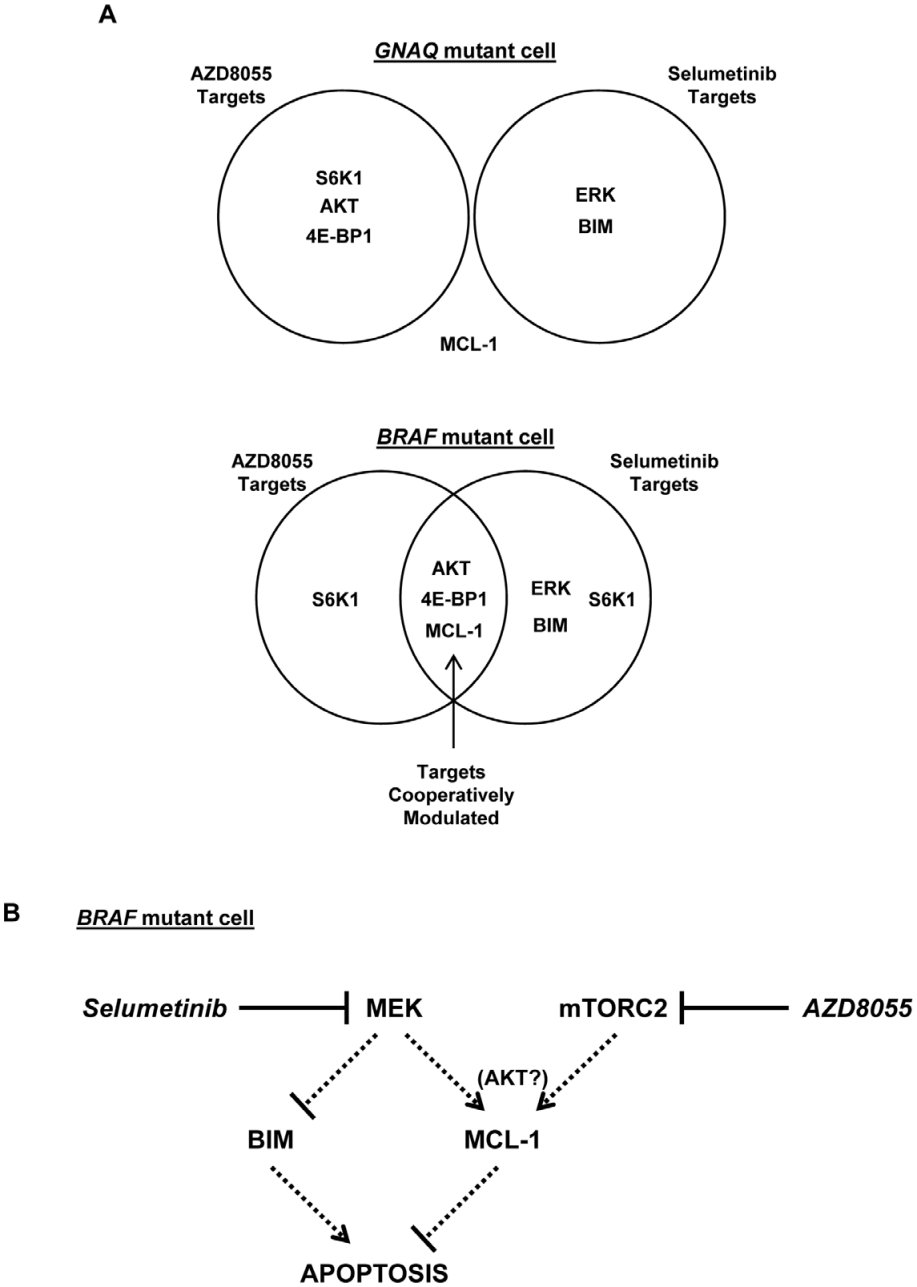
Diagrammatic representation of signaling proteins and pathways modulated by AZD8055 and selumetinib. *A,* This study revealed that AZD8055 and selumetinib impacted the regulation of several signaling proteins differently in *GNAQ* mutant versus *BRAF* mutant cells. The proteins AKT, 4E-BP1, and MCL-1 were cooperatively regulated by the AZD8055/selumetinib combination in *BRAF* mutant cells, while none of the candidate targets examined were found to be cooperatively regulated in *GNAQ* mutant cells. *B,* Diagram of the signaling pathways impacted by selumetinib and AZD8055 to induce apoptosis in *BRAF* mutant cells. Our data suggests that BIM upregulation and MCL-1 downregulation are necessary for apoptosis achieved with dual pathway inhibition. While BIM was induced by selumetinib, MCL-1 was decreased by combined mTORC2 inhibition and MEK inhibition by AZD8055 and selumetinib, respectively, possibly through cooperative AKT inhibition. The dotted arrows indicate that drug mediated inhibition of MEK and mTORC2 likely influence BIM and MCL-1 through molecular intermediates.

Further elucidation of the differences in drug-induced outcomes for *GNAQ* and *BRAF* mutant uveal melanomas revealed that AKT, 4E-BP1, and MCL-1 were cooperatively regulated by the AZD8055/selumetinib combination only in *BRAF* mutant cells and not *GNAQ* cells (**Figure 8A**), suggesting these as candidate targets responsible for the distinct apoptotic outcomes observed. In fact, activation of RTKs (including IGF-1R), AKT, and 4E-BP1 have each been implicated as critical mechanisms of resistance to MAPK pathway inhibition in other systems [23], [31], [39]–[41]. Establishing apoptosis as an important cellular outcome that correlates to *in vivo* anti-tumor effects in our model allowed us to critically evaluate whether the biochemical modulation of any of these molecules are truly critical for the anti-tumor effects elicited with dual pathway inhibition. Our data revealed that while 4E-BP1 is cooperatively regulated by AZD8055/selumetinib, this is not critical for the induction of apoptosis in *BRAF* mutant uveal melanoma cells. Selumetinib suppression of AZD8055-mediated activation of the IGF-1R/AKT axis alone is also not sufficient to induce apoptosis in these cells. Instead, AZD8055 suppression of mTORC2 cooperates with selumetinib to reduce MCL-1 protein expression, a change that in combination with selumetinib-mediated induction of BIM, is essential for combination-induced apoptosis (**Figure 8B**). In the context of recent data arguing that the superior *in vitro* effects of ATP-competitive mTOR inhibitors over rapamycin are related primarily to more effective mTORC1 targeting rather than mTORC2 inhibition [17]–[19], the findings presented here also argue that mTORC2 may still be a relevant therapeutic target.

In *EGFR* mutant lung cancer cells, inhibition of PI3K/mTOR and MEK were directly linked to MCL-1 downregulation and BIM upregulation, respectively [27]. In this study, MCL-1 downregulation correlated with cooperative inhibition of AKT phosphorylation in BRAF cells, suggesting that concomitant MEK and AKT inhibition may be essential for altering both BIM and MCL-1 levels to induce apoptosis in these cells as well (**Figure 8B**). We also observed that dual MEK and AKT inhibition can be achieved by combining selumetinib with the IGF-1R inhibitor NVP-AEW541, and also resulted in decreased MCL-1, increased BIM, and induction of apoptosis (**Figure S7**). Hence, different targeted approaches may achieve the dual pathway inhibition that can impact BIM/MCL-1 levels and induce apoptosis in *BRAF* mutant cells.

What remains unknown is how differences in uveal melanoma PTEN status may influence drug susceptibility independent of *GNAQ* and *BRAF* mutation status. We did observe that *BRAF* mutant cell lines had overall less *PTEN* protein expression compared to WT or GNAQ cells, though this did not result in baseline differences of AKT or S6K1 phosphorylation (**Figure S8A**). Since *PTEN* null uveal melanoma cell lines are not currently available [42], the contribution of *PTEN* status to drug susceptibility can not be fully addressed. We did investigate how siRNA-mediated depletion of *PTEN* expression in the GNAQ cell line 92.1 impacted drug-induced apoptosis and found this did not change the drug effects upon the relevant signaling pathways or MCL-1 levels (**Figure S8B**). Though there was also no statistically significant increase in apoptosis with PTEN siRNA compared to control siRNA transfected cells, we did observe a very small trend towards higher apoptosis with the selumetinib/AZD8055 combination in cells with suppressed PTEN expression (**Figure S8B**). We concluded that while PTEN status may modestly influence the induction of apoptosis, it does so in a manner distinct from the MCL-1 dependent mechanism we have delineated for *BRAF* mutant cells, though further study regarding the contribution of PTEN status apart from *BRAF or GNAQ* mutations is warranted.

One question stemming from this study is how applicable the efficacy of combined mTOR and MEK inhibition may be for other *BRAF* mutant malignancies. We have evaluated the AZD8055/selumetinib combination in cutaneous melanomas and have found that the combination preferentially induced apoptosis in most *BRAF* mutant cell lines (3 out of 4 lines tested), but not *N-RAS* mutant cells or cells lacking these mutations (**Figure S9**), supporting observations from Gopal et. al. [31]. For *GNAQ* mutant cells, further study is warranted to develop more effective *GNAQ* targeting strategies in order to achieve better therapeutic efficacy.

## Methods

### Chemicals

Selumetinib and AZD8055 were supplied by AstraZeneca (Wilmington, DE, USA) and dissolved in DMS0 at 10 mM and stored at −20°C. NVP-AEW541 was purchased from Caymen Chemical (Ann Arbor, MI, USA) and dissolved in PBS at 5 mM and stored at −20°C. Rapamycin was purchased from Calbiochem (Gibbstown, NJ, USA) and dissolved at 5 mM in DMSO and stored at −20°C.

### Cell Culture

OCM1A [43] and 92.1 [44] were provided by Dr. William Harbour (Washington University, St. Louis, MO). Mel270 [45] was provided by Dr. Bruce Ksander (Harvard Medical School, Boston, MA, USA). OCM3 [46], [47] and Mel290 [48] were provided by Dr. Robert Folberg (University of Illinois, Chicago, IL). C918 [49] was provided by Dr. David H. Abramson (MSKCC, New York, NY). All uveal melanoma cell lines were maintained in RPMI1640 supplemented with heat-inactivated 10% fetal bovine serum plus penicillin and streptomycin.

### Cell Viability Assays and Combination Index Analysis

Cell viability assays were performed with Dojindo Molecular Technologies (Rockville, MD, USA) per manufacturer’s instructions. For the combination index (CI) analysis, dose-effect curve parameters for both AZD8055 and selumetinib were used for the automated calculation of CI values conducted by the CompuSyn software (ComboSyn, Paramus, NJ, USA) [50]. Fa-CI plots (Chou-Talalay plots) were generated where Fa is the fraction affected (Fa =  percentage of inhibition relative to vehicle control/100) [50]. CompuSyn software was also utilized to calculate the 50% growth inhibitory values (GI50s).

### Flow Cytometry

Bi-parameter flow cytometry analysis for DNA content (detected with propidium iodide) and another maker (including MPM-2 or MCL-1) was performed as previously described [51].

### Western Blots

Cell lysates and Western blots were performed as previously described [52]. The antibodies for phosphorylated ERK 1/2 (Y204) and Ku70 were obtained from Santa Cruz Biotechnology (Santa Cruz, CA). All the other antibodies were obtained from Cell Signaling Technology (Boston, MA).

### Gene Silencing

Experiments with small interfering RNAs (siRNA) were performed as previously described [52]. Pooled siRNA constructs targeting 4E-BP1 (L-003005), RICTOR (L-016984), and RAPTOR (L-004107) were purchased from Dharmacon (ON-TARGET plus SMART pool; Lafayette, CO, USA). Pooled unrelated control (sc-37007), GNAQ (sc-35429), and MCL-1 (sc-35877) siRNAs were also purchased from Santa Cruz Biotechnology (Santa Cruz, CA, USA).

### Cap-Binding Assay

Assays with m^7^ GTP sepharose beads (GE Healthcare) were performed as previously described [23]. Cells were lysed in RIPA buffer. 30 µl of 7-methyl GTP-Sepharose beads (GE healthcare) were washed twice in 500 µl of RIPA buffer. 150 µg of each lysate was added to separately prepared beads and incubated at 4°C on a rotator for two hours. The complexes were centrifuged at 2000 rpm and washed three times with RIPA buffer. 50 µl of NuPAGE LDS sample buffer (Invitrogen) were added to the samples and then boiled at 95°C for 5 minutes. Samples were then analyzed by Western blot as described above.

### MCL-1 Overexpression

Cells were transiently transfected with an MCL-1 expression plasmid as previously described [53]. The MCL-l expression plasmid was provided by Dr. Hannah Rabinowich and Dr. Leslie Goldstein (University of Pittsburgh School of Medicine, Pittsburgh, PA).

### Xenograft Studies

8-week-old nu/nu SCID male mice bearing subcutaneously injected OCM1A or 92.1 tumors (7 mice/cohort) of ∼100 mm^3^ diameter were treated orally (p.o.) with vehicle, AZD8055 (20 mg/kg/d), and/or selumetinib (25 mg/kg/d) as single agents and in combination, 5 days/week for 3 weeks. After the fifth treatment, two animals from each cohort were sacrificed and the tumors were assessed by hematoxylin & eosin staining (H&E), Ki67 staining, terminal deoxynucleotidyl transferase dUTP nick end labeling (TUNEL) staining, and Western blot as previously described [53]. Mice in the control groups were treated with a 0.5% HPMC +0.1% Tween 80 control solution. Tumors were measured every 2 to 3 days with calipers and tumor volumes were calculated and expressed in cubic millimeter and calculated using the formula p/6 × (large diameter) × (small diameter). Toxicity was monitored by weight loss. At the end of the study, mice were euthanized by CO2 asphyxiation. Experiments were carried out under institutional guidelines addressing the proper and humane use of animals. The Memorial Sloan-Kettering Cancer Center Institutional Animal Care and Use Committee and Research Animal Resource Center specifically approved this study. The study also complied with the Principles of Laboratory Animal Care (NIH Publication No. 85–23, released 1985). All efforts were made to minimize suffering.

### Statistical Analysis

All *in vitro* experiments were carried out at least 2–3 times. Standard error was calculated as the standard deviation divided by the square root of the number of samples. For *in vivo* studies, p-values were calculated using an exact version of the Wilcoxon Rank Sum test. We selected p values ≤0.05 being statistically significant. Tumor volumes were compared between groups of mice at various time points.

## Supporting Information

Figure S1 **GI50 values for uveal melanoma cell lines treated with various concentrations of selumetinib and AZD8055.** *A,* Cells were treated with selumetinib at various concentrations (0, 20, 50, 100, 1000, 5000 nM) for 96 hours and treated wells then analyzed by viability assay. GI50 values were determined utilizing CompuSyn. *B,* Cells were treated with AZD8055 at various concentrations (0, 20, 50, 100, 1000 nM) for 96 hours and analysis/calculations for viability and GI50s were performed as described in *A*.
(TIF)

Figure S2 **Impact of AZD8055 and selumetinib upon MAPK and AKT/mTOR pathway signaling.** *A,* Western blot of S6K1, AKT, and ERK phosphorylation following AZD8055 for 24 hours. *B,* Western blot of ERK and AKT phosphorylation following exposure to selumetinib for 24 hours. *C,* AKT phosphorylation over time with 100 nM AZD8055. *D,* ERK phosphorylation over time with 1000 nM selumetinib. *Interpretation:* For AZD8055, 100 nM was the lowest concentration to suppress phosphorylation of the mTORC1 substrate S6 Kinase 1 (S6K1) at 24 hours in all the cell lines (**Figure S2A**). 100 nM AZD8055 impacted the phosphorylation of mTORC2 substrate AKT (at S473) more variably as BRAF and GNAQ, but not WT, cells retained phosphorylated AKT at levels approximating those in vehicle-treated cells (**Figure S2A**). This was due to rebound AKT phosphorylation: 100 nM AZD8055 inhibited AKT phosphorylation at 2 hours in all three genotypes and by 24 hours AKT phosphorylation rebounded only in BRAF and GNAQ cells (**Figure S2C**). This implies that a PDK2 other than mTORC2 may be re-activated after prolonged AZD8055 exposure. Interestingly, higher concentrations of AZD8055 inhibited AKT phosphorylation (**Figure S2A**), which we concluded represented off-target activity given 1) effective mTORC2 inhibition in all the cell lines at 2 hours with 100 nM AZD8055, and 2) discordance between inhibition of AKT and S6K1 phosphorylation in BRAF cells (a discrepancy not observed in WT cells), confirming that mTOR inhibition occurs at concentrations as low as 20 nM in BRAF cells. While rebound AKT phosphorylation was accompanied by rebound S6K1 phosphorylation in the *GNAQ* mutant cell line, this was not observed in the *BRAF* mutant cell line, suggesting different mechanism of rebound phosphorylation may exist. For selumetinib, 1000 nM was the lowest concentration that inhibited phosphorylation of the MEK substrates ERK1/2 at 2 hours, but rebound ERK phosphorylation occurred preferentially in WT cells after 24 hours (**Figure S2D**).
(TIF)

Figure S3 **Immunohistochemistry staining for TUNEL and Ki67 in** ***BRAF*** **and** ***GNAQ*** **mutant xenograft models.** *A* and *B,* After the fifth drug(s) or vehicle dose, two animals from each cohort were sacrificed and the tumors were assessed for TUNEL and Ki67 staining. See *Methods* for the details of this analysis.
(TIF)

Figure S4 **NVP-AEW541 inhibits IGF-1R phosphorylation.** *A,* Cells were serum-starved for 24 hours and then exposed to vehicle or 1000 nM NVP-AEW541 either in serum-free conditions or in the presence of 5 µg/ml of IGF-1 ligand. Treatment was for 10 minutes. Cell lysates were created and Western blot was then performed. *B,* Cells were treated with the indicated vehicles or drugs for 24 hours and then cell lysates were created for RTK antibody array blots. Drug concentrations used: 100 nM AZD8055, 1000 nM selumetinib, and 1000 nM NVP-AEW541.
(TIF)

Figure S5 **Bi-parametric flow cytometry for MCL-1 transfected OCM1A cells.** Cells were transiently transfected with an *MCL-1* cDNA expression plasmid under the control of a constitutively active viral promoter or an empty vector for 48 hours. Cells were then treated with vehicle, 100 nM AZD8055, 1000 nM selumetinib, or the combination for 24 hours and then analyzed by bi-parametric flow cytometry for DNA content and MCL-1 expression levels. *Red circle*, sub-G1 fraction in both the low- and high- MCL-1 expressing populations.
(TIF)

Figure S6 **AZD8055/selumetinib cooperatively downregulates MCL-1 and upregulates BIM in an** ***in vivo*** **BRAF mutant xenograft model.** With the xenograft experiment performed upon the *BRAF* mutant cell line OCM1A described in **Figure 2**, two animals from each treatment cohort were sacrificed after the fifth drug administration and tumors were flash frozen and processed for immunoblot analysis of BIM and MCL-1 levels (Ku70 was utilized as a loading control). Each lane represents a separate animal.
(TIF)

Figure S7 **Dual pathway inhibition with selumetinib and the IGF-1R inhibitor NVP-AEW541 induces apoptosis in OCM1A cells.** Cells were treated with vehicle, 1000 nM selumetinib, 100 nM AZD8055, 1000 nM NVP-AEW541, the selumetinib/AZD8055 combination, or the selumetinib/NVP-AEW541 combination. After 24 hours, cell lysates were created and Western blot was performed. After 48 hours, flow cytometry for DNA content was performed and the percentage of sub-G1 cells was quantified. Results reflect triplicate samples for each condition. The selumetinib/NVP-AEW541 combination achieved the same dual pathway inhibition (with suppression of AKT, S6K1, and ERK phosphorylation), cooperative MCL-1 downregulation, BIM upregulation, and induction of apoptosis that was observed with the selumetinib/AZD8055 combination. See the RTK array blots in **Figure S4B** confirming that NVP-AEW541 inhibits IGF-1R phosphorylation alone and in combination with selumetinib in these conditions. *Error bars, SE.*
(TIF)

Figure S8 **PTEN levels and the susceptibility to the selumetinib/AZD8055 combination.** *A,* Protein expression of PTEN and several other phospho-proteins in the six uveal and two cutaneous melanoma (PTEN-negative Mel 133 and PTEN-positive Mel 32) cell lines was examined by Western blot. *B,* siRNA mediated suppression of *PTEN* in the GNAQ cell line 92.1 does not significantly change the impact of the selumetinib/AZD8055 combination upon the targeted pathways or apoptosis. Cells were transfected with pooled siRNA constructs targeting *PTEN* or unrelated control constructs for 48 hours and then treated with the indicated drugs for 24 hours. Western blots were then performed. After 48 hours of drug treatment, flow cytometry for DNA content was performed and the percentage of sub-G1 cells was quantified. Results reflect triplicate samples for each condition. *Error bars, SE.*
(TIF)

Figure S9 **AZD8055/selumetinib induces apoptosis preferentially in** ***BRAF*** **mutant cutaneous melanomas.** A panel of cutaneous melanoma cell lines of the indicated genotypes were treated with the indicated drugs for 72 hours (with the exception with MEL 267 which was treated for 48 hours) and then assayed by flow cytometry for DNA content in order to determine the percentage of cells with sub-G1 DNA, indicative of apoptosis. *WT,* defined as cells lacking *BRAF, RAS,* or any other known pathway mutations; *N-RAS mutation*, MEL 30 has a Q61K mutation and MEL 103 has a Q61R mutation in N-RAS; *BRAF mutation*, V600E.
(TIF)

Table S1 **List of the uveal melanoma cell lines utilized in this study.** The gene mutations for each cell line listed were previously described/discovered by other groups. We also analyzed the cell lines on a mass spectrometry platform to assess for other hotspot gene mutations. Some of the relevant mutations tested include: *AKT1* (E17K, R1114, Q1303, E1306*/K, E1308*/K, E1309*/K, E1322*, Q1338*, Q1367*, Q1367H, Q1378*, Q1379*, Q1406*, Q1429*, R1450*, S1465fs*3), *PIK3CA* (E542Q), *ARAF*, *BRAF*, *EGFR*, ERBB2, *FGFR1-4, KIT, PDGFR A/B, KRAS, NRAS, HRAS,* and *MEK1*.
(TIF)
