# Supplementary figures and images for: Impact of Combined mTOR and MEK Inhibition in Uveal Melanoma Is Driven by Tumor Genotype
Source: PLoS One. 2012 Jul 10;7(7):e40439. doi: 10.1371/journal.pone.0040439 (PMC3393714; doi:10.1371/journal.pone.0040439)

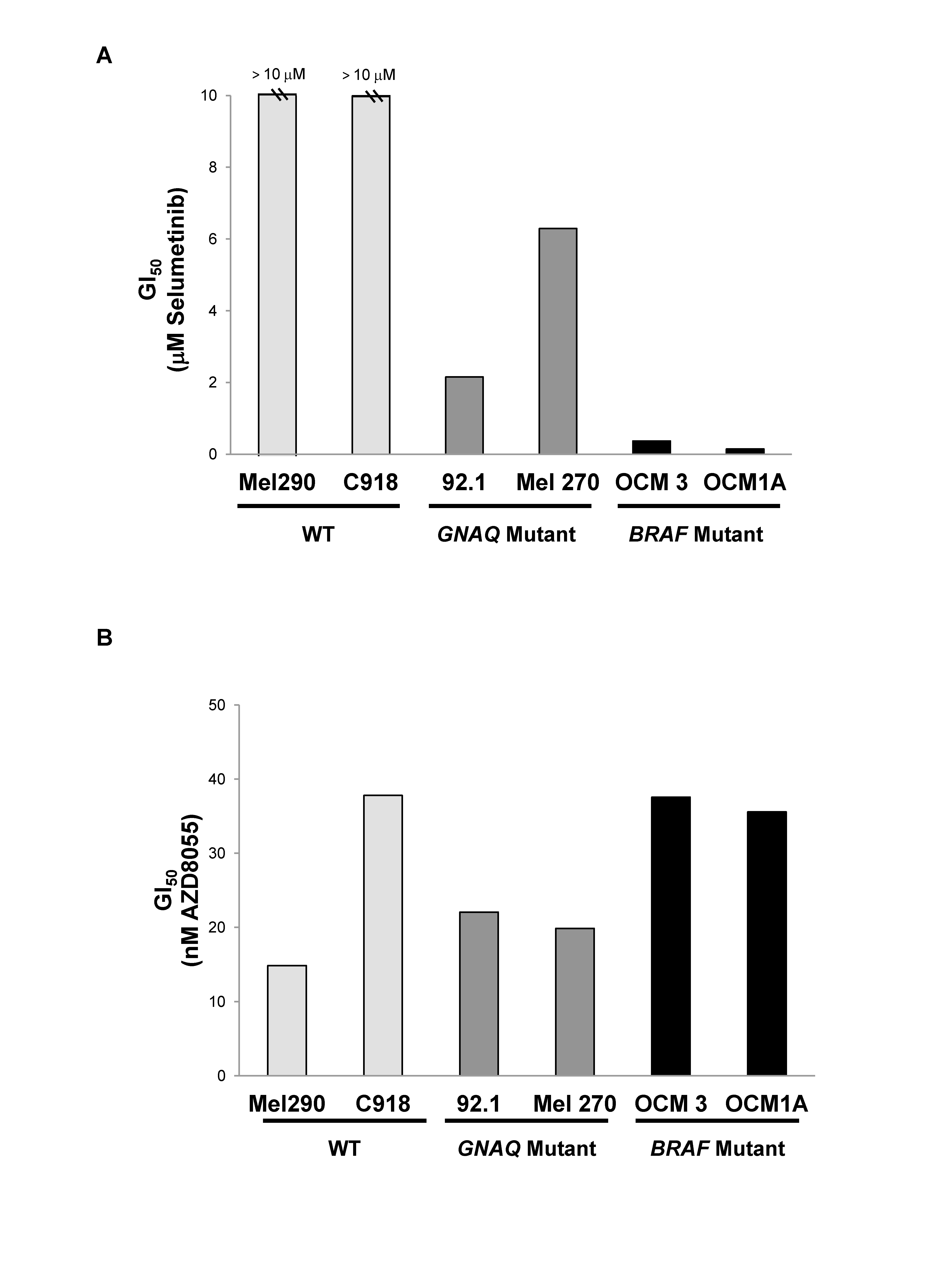

Supplement: Figure S1 — GI50 values for uveal melanoma cell lines treated with various concentrations of selumetinib and AZD8055. A, Cells were treated with selumetinib at various concentrations (0, 20, 50, 100, 1000, 5000 nM) for 96 hours and treated wells then analyzed by viability assay. GI50 values were determined utilizing CompuSyn. B, Cells were treated with AZD8055 at various concentrations (0, 20, 50, 100, 1000 nM) for 96 hours and analysis/calculations for viability and GI50s were performed as described in A. (TIF) [file pone.0040439.s001.tif]

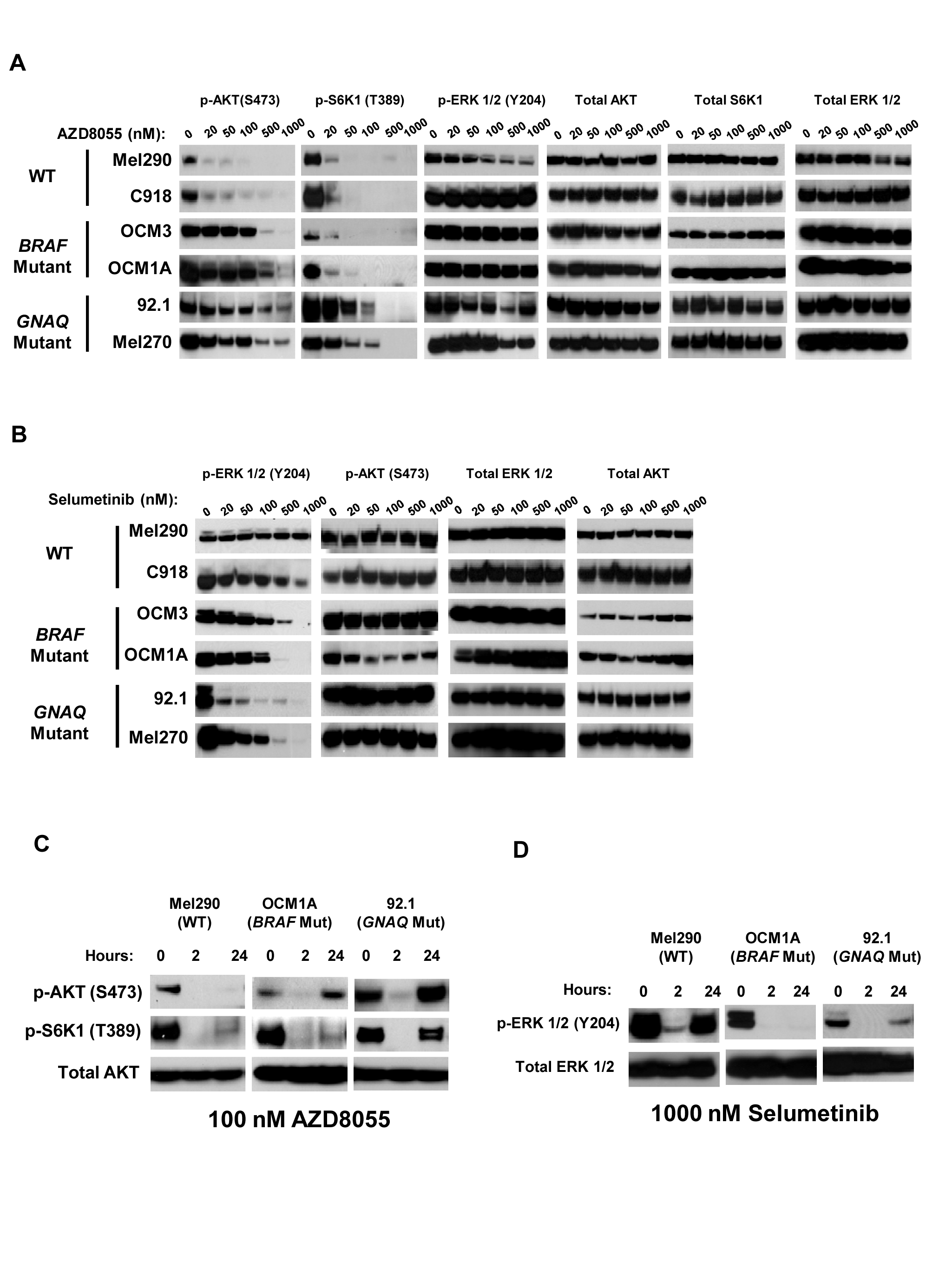

Supplement: Figure S2 — Impact of AZD8055 and selumetinib upon MAPK and AKT/mTOR pathway signaling. A, Western blot of S6K1, AKT, and ERK phosphorylation following AZD8055 for 24 hours. B, Western blot of ERK and AKT phosphorylation following exposure to selumetinib for 24 hours. C, AKT phosphorylation over time with 100 nM AZD8055. D, ERK phosphorylation over time with 1000 nM selumetinib. Interpretation: For AZD8055, 100 nM was the lowest concentration to suppress phosphorylation of the mTORC1 substrate S6 Kinase 1 (S6K1) at 24 hours in all the cell lines (Figure S2A). 100 nM AZD8055 impacted the phosphorylation of mTORC2 substrate AKT (at S473) more variably as BRAF and GNAQ, but not WT, cells retained phosphorylated AKT at levels approximating those in vehicle-treated cells (Figure S2A). This was due to rebound AKT phosphorylation: 100 nM AZD8055 inhibited AKT phosphorylation at 2 hours in all three genotypes and by 24 hours AKT phosphorylation rebounded only in BRAF and GNAQ cells (Figure S2C). This implies that a PDK2 other than mTORC2 may be re-activated after prolonged AZD8055 exposure. Interestingly, higher concentrations of AZD8055 inhibited AKT phosphorylation (Figure S2A), which we concluded represented off-target activity given 1) effective mTORC2 inhibition in all the cell lines at 2 hours with 100 nM AZD8055, and 2) discordance between inhibition of AKT and S6K1 phosphorylation in BRAF cells (a discrepancy not observed in WT cells), confirming that mTOR inhibition occurs at concentrations as low as 20 nM in BRAF cells. While rebound AKT phosphorylation was accompanied by rebound S6K1 phosphorylation in the GNAQ mutant cell line, this was not observed in the BRAF mutant cell line, suggesting different mechanism of rebound phosphorylation may exist. For selumetinib, 1000 nM was the lowest concentration that inhibited phosphorylation of the MEK substrates ERK1/2 at 2 hours, but rebound ERK phosphorylation occurred preferentially in WT cells after 24 hours (Figure S2D). (TIF) [file pone.0040439.s002.tif]

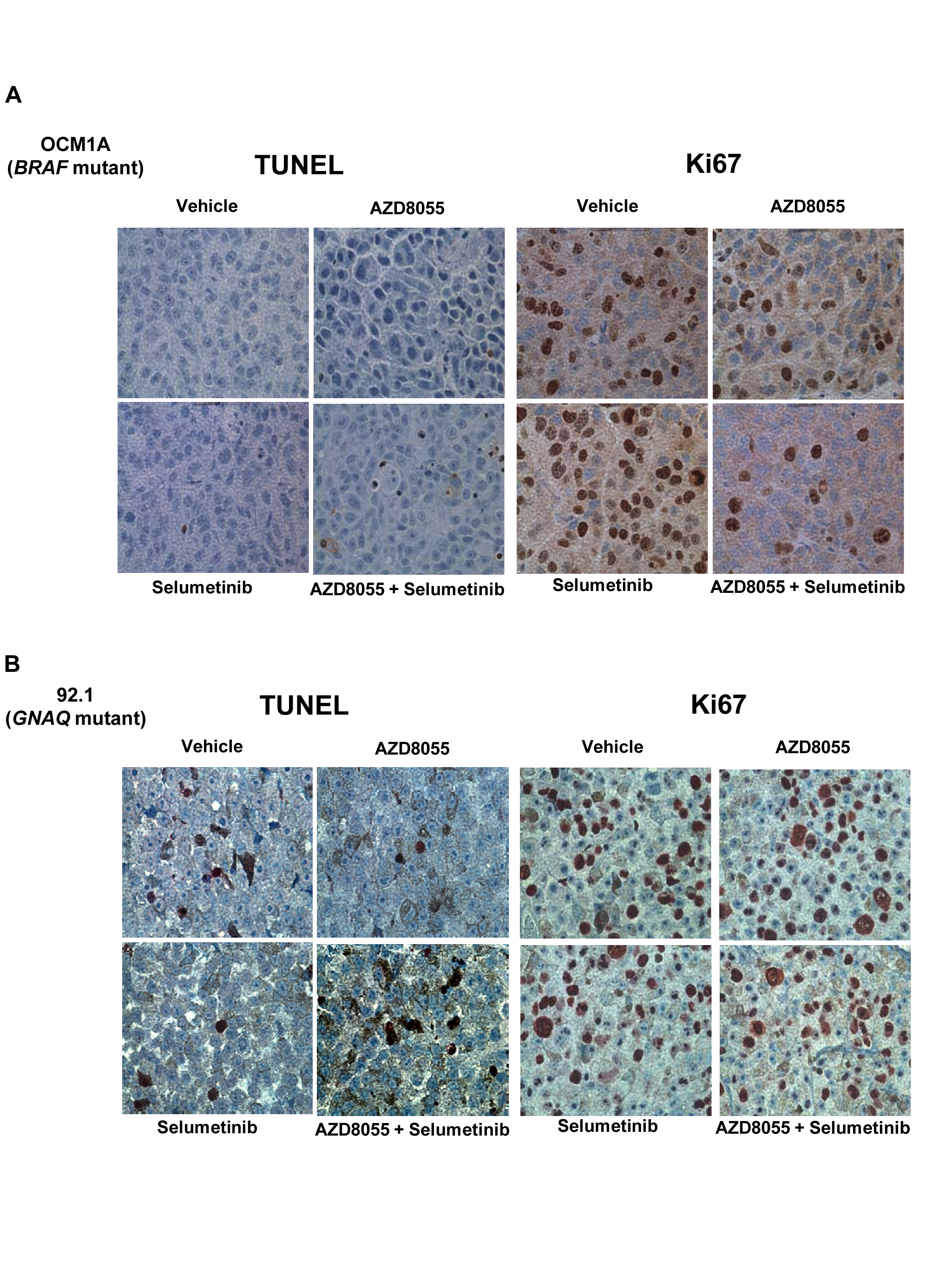

Supplement: Figure S3 — Immunohistochemistry staining for TUNEL and Ki67 in BRAF and GNAQ mutant xenograft models. A and B, After the fifth drug(s) or vehicle dose, two animals from each cohort were sacrificed and the tumors were assessed for TUNEL and Ki67 staining. See Methods for the details of this analysis. (TIF) [file pone.0040439.s003.tif]

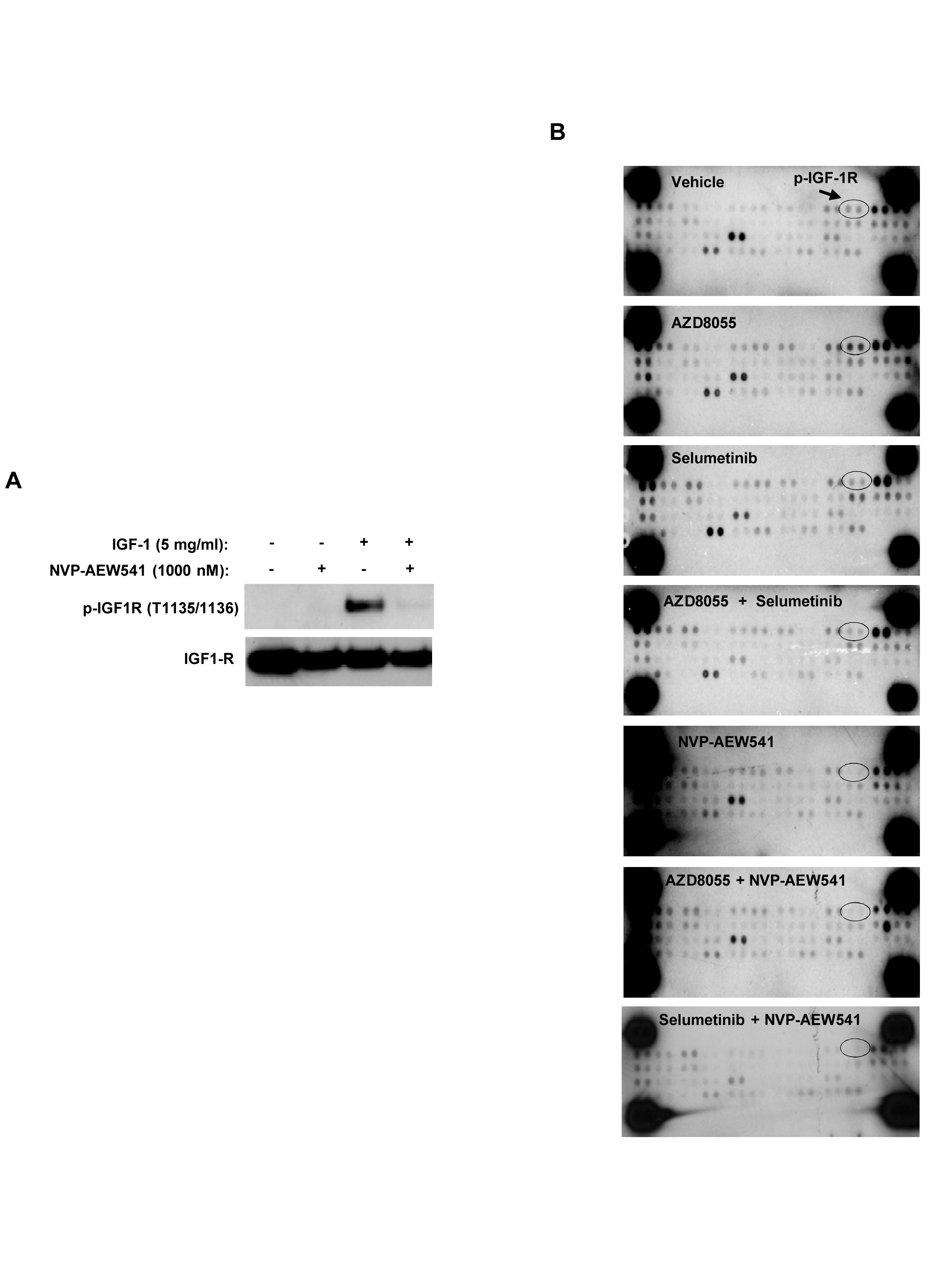

Supplement: Figure S4 — NVP-AEW541 inhibits IGF-1R phosphorylation. A, Cells were serum-starved for 24 hours and then exposed to vehicle or 1000 nM NVP-AEW541 either in serum-free conditions or in the presence of 5 µg/ml of IGF-1 ligand. Treatment was for 10 minutes. Cell lysates were created and Western blot was then performed. B, Cells were treated with the indicated vehicles or drugs for 24 hours and then cell lysates were created for RTK antibody array blots. Drug concentrations used: 100 nM AZD8055, 1000 nM selumetinib, and 1000 nM NVP-AEW541. (TIF) [file pone.0040439.s004.tif]

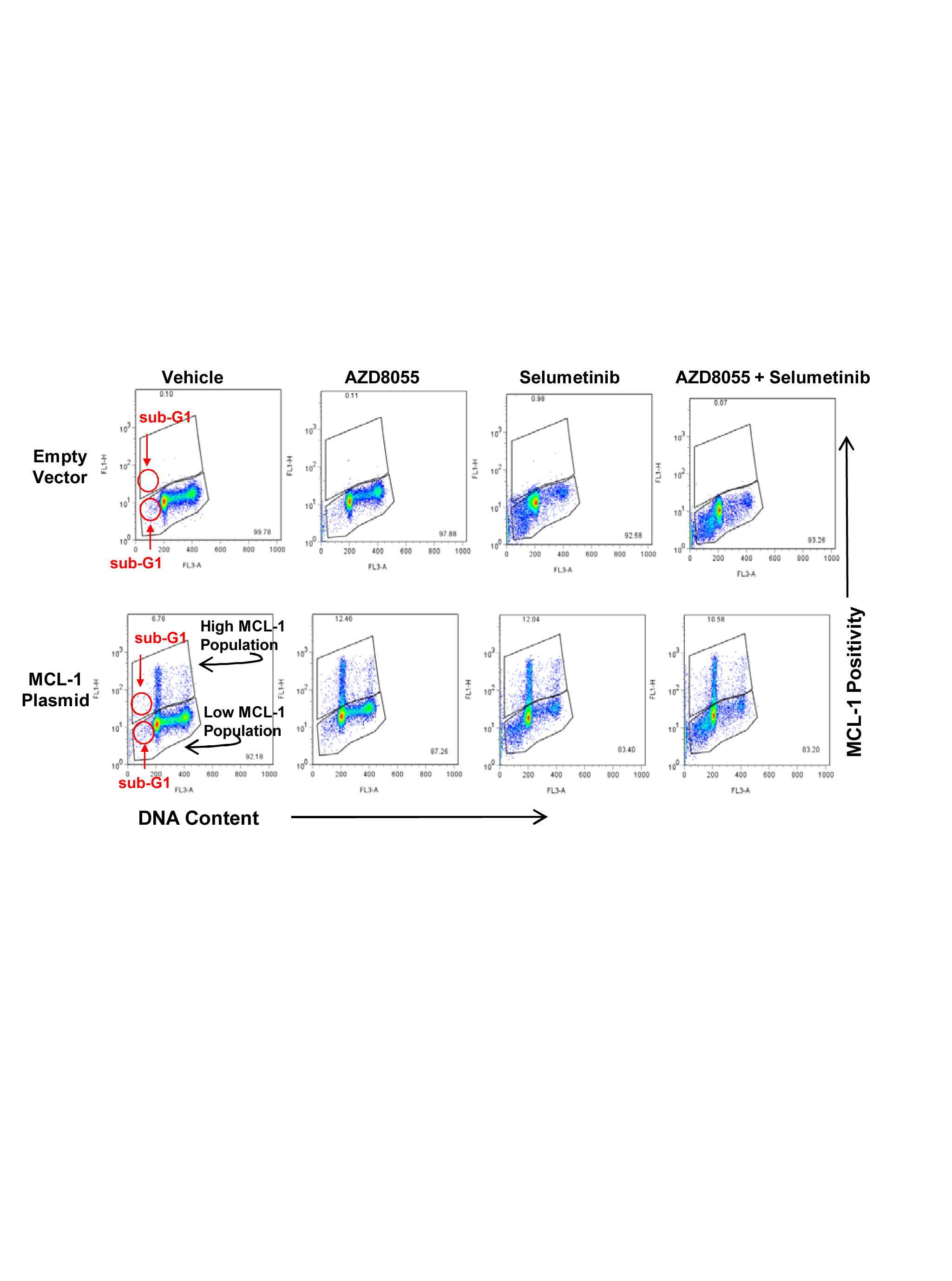

Supplement: Figure S5 — Bi-parametric flow cytometry for MCL-1 transfected OCM1A cells. Cells were transiently transfected with an MCL-1 cDNA expression plasmid under the control of a constitutively active viral promoter or an empty vector for 48 hours. Cells were then treated with vehicle, 100 nM AZD8055, 1000 nM selumetinib, or the combination for 24 hours and then analyzed by bi-parametric flow cytometry for DNA content and MCL-1 expression levels. Red circle, sub-G1 fraction in both the low- and high- MCL-1 expressing populations. (TIF) [file pone.0040439.s005.tif]

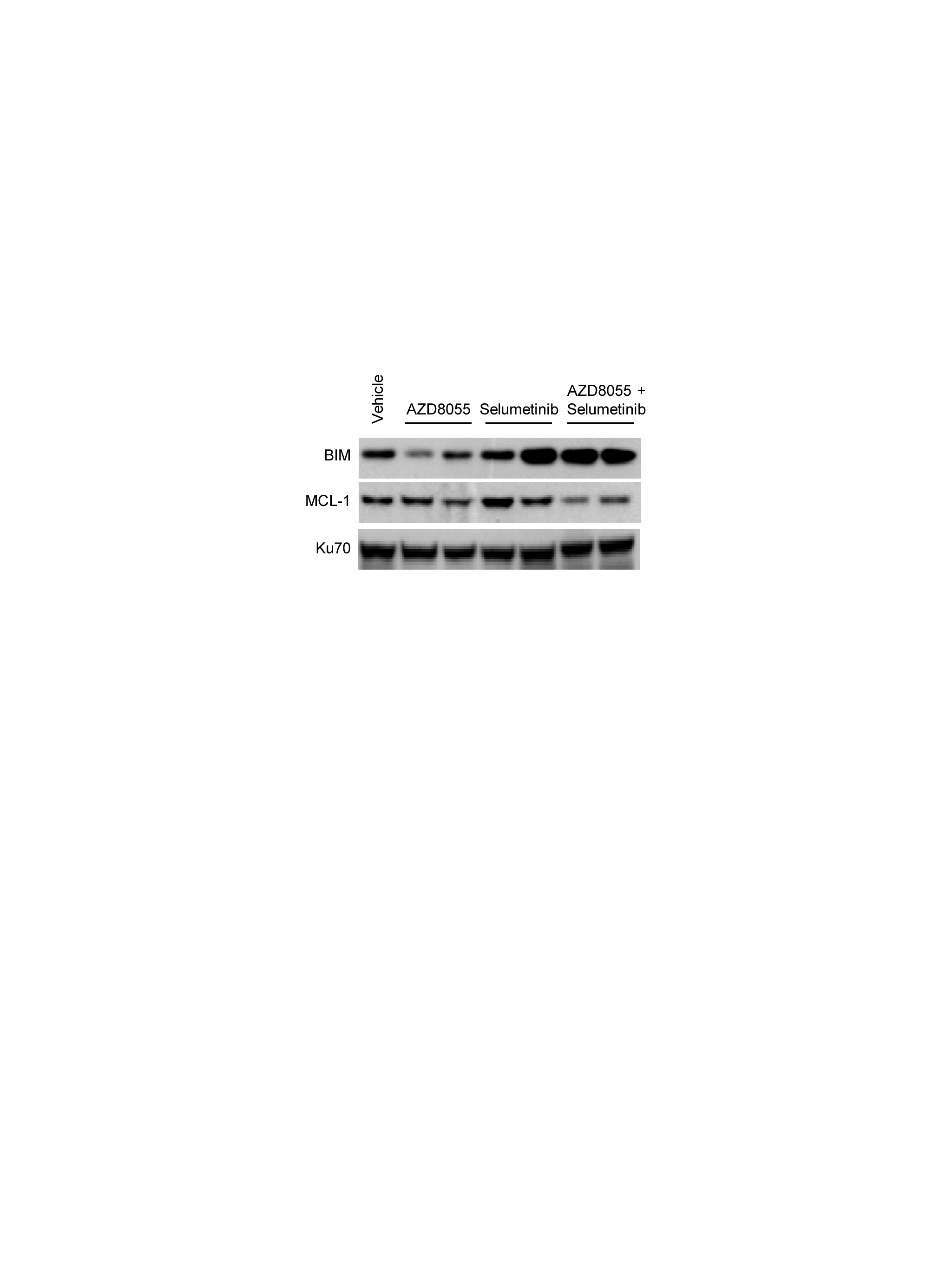

Supplement: Figure S6 — AZD8055/selumetinib cooperatively downregulates MCL-1 and upregulates BIM in an in vivo BRAF mutant xenograft model. With the xenograft experiment performed upon the BRAF mutant cell line OCM1A described in Figure 2 , two animals from each treatment cohort were sacrificed after the fifth drug administration and tumors were flash frozen and processed for immunoblot analysis of BIM and MCL-1 levels (Ku70 was utilized as a loading control). Each lane represents a separate animal. (TIF) [file pone.0040439.s006.tif]

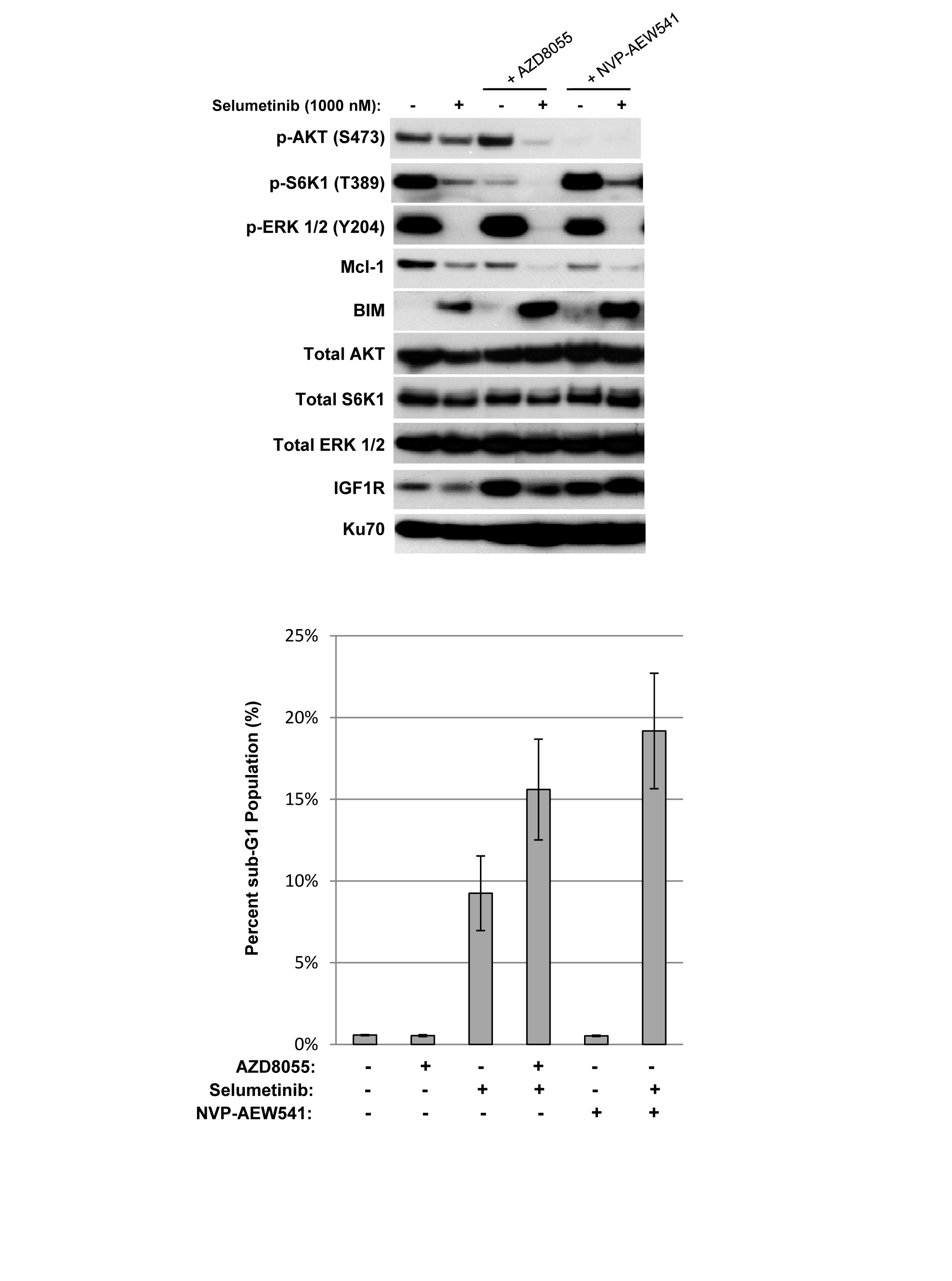

Supplement: Figure S7 — Dual pathway inhibition with selumetinib and the IGF-1R inhibitor NVP-AEW541 induces apoptosis in OCM1A cells. Cells were treated with vehicle, 1000 nM selumetinib, 100 nM AZD8055, 1000 nM NVP-AEW541, the selumetinib/AZD8055 combination, or the selumetinib/NVP-AEW541 combination. After 24 hours, cell lysates were created and Western blot was performed. After 48 hours, flow cytometry for DNA content was performed and the percentage of sub-G1 cells was quantified. Results reflect triplicate samples for each condition. The selumetinib/NVP-AEW541 combination achieved the same dual pathway inhibition (with suppression of AKT, S6K1, and ERK phosphorylation), cooperative MCL-1 downregulation, BIM upregulation, and induction of apoptosis that was observed with the selumetinib/AZD8055 combination. See the RTK array blots in Figure S4B confirming that NVP-AEW541 inhibits IGF-1R phosphorylation alone and in combination with selumetinib in these conditions. Error bars, SE. (TIF) [file pone.0040439.s007.tif]

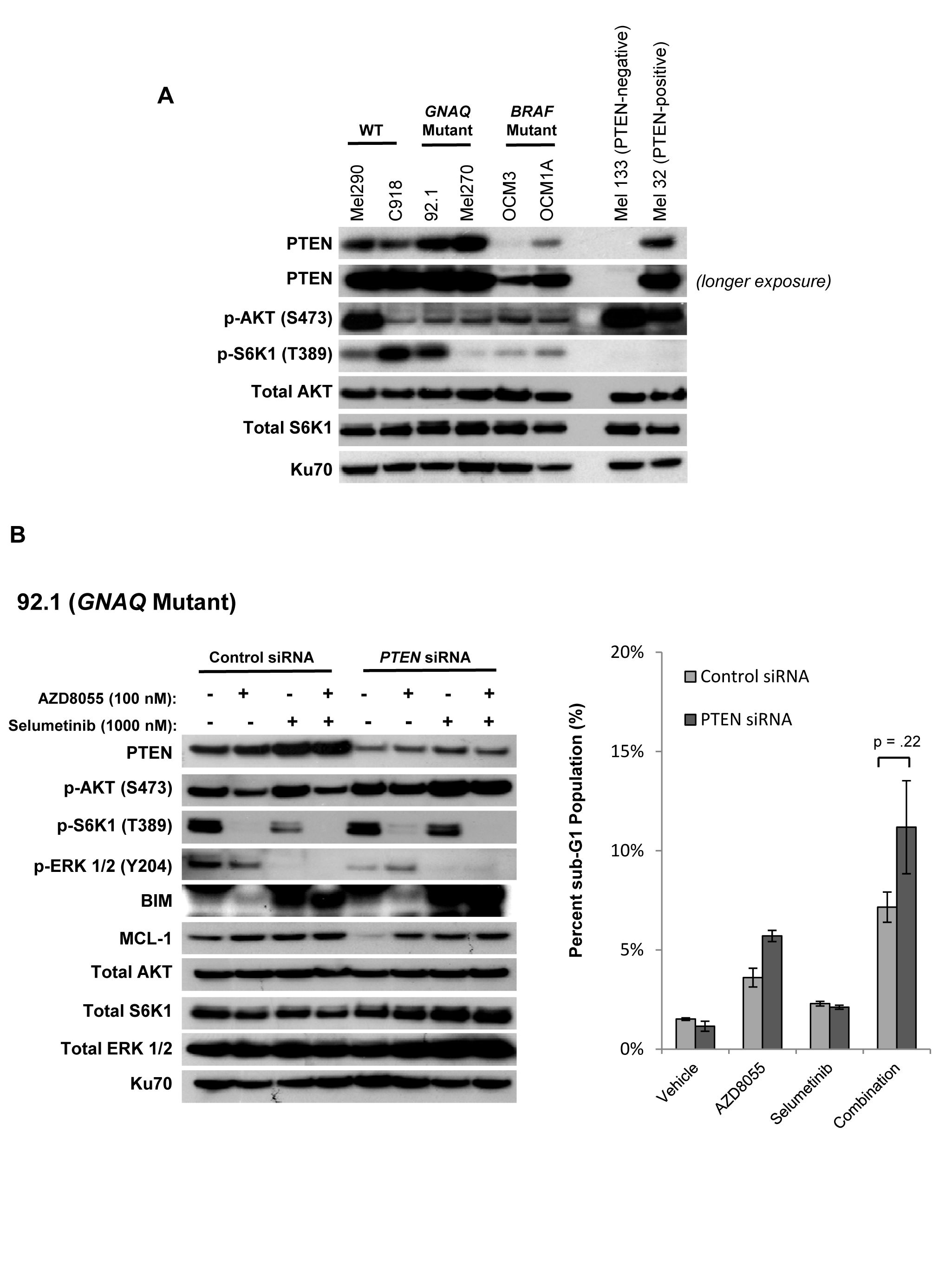

Supplement: Figure S8 — PTEN levels and the susceptibility to the selumetinib/AZD8055 combination. A, Protein expression of PTEN and several other phospho-proteins in the six uveal and two cutaneous melanoma (PTEN-negative Mel 133 and PTEN-positive Mel 32) cell lines was examined by Western blot. B, siRNA mediated suppression of PTEN in the GNAQ cell line 92.1 does not significantly change the impact of the selumetinib/AZD8055 combination upon the targeted pathways or apoptosis. Cells were transfected with pooled siRNA constructs targeting PTEN or unrelated control constructs for 48 hours and then treated with the indicated drugs for 24 hours. Western blots were then performed. After 48 hours of drug treatment, flow cytometry for DNA content was performed and the percentage of sub-G1 cells was quantified. Results reflect triplicate samples for each condition. Error bars, SE. (TIF) [file pone.0040439.s008.tif]

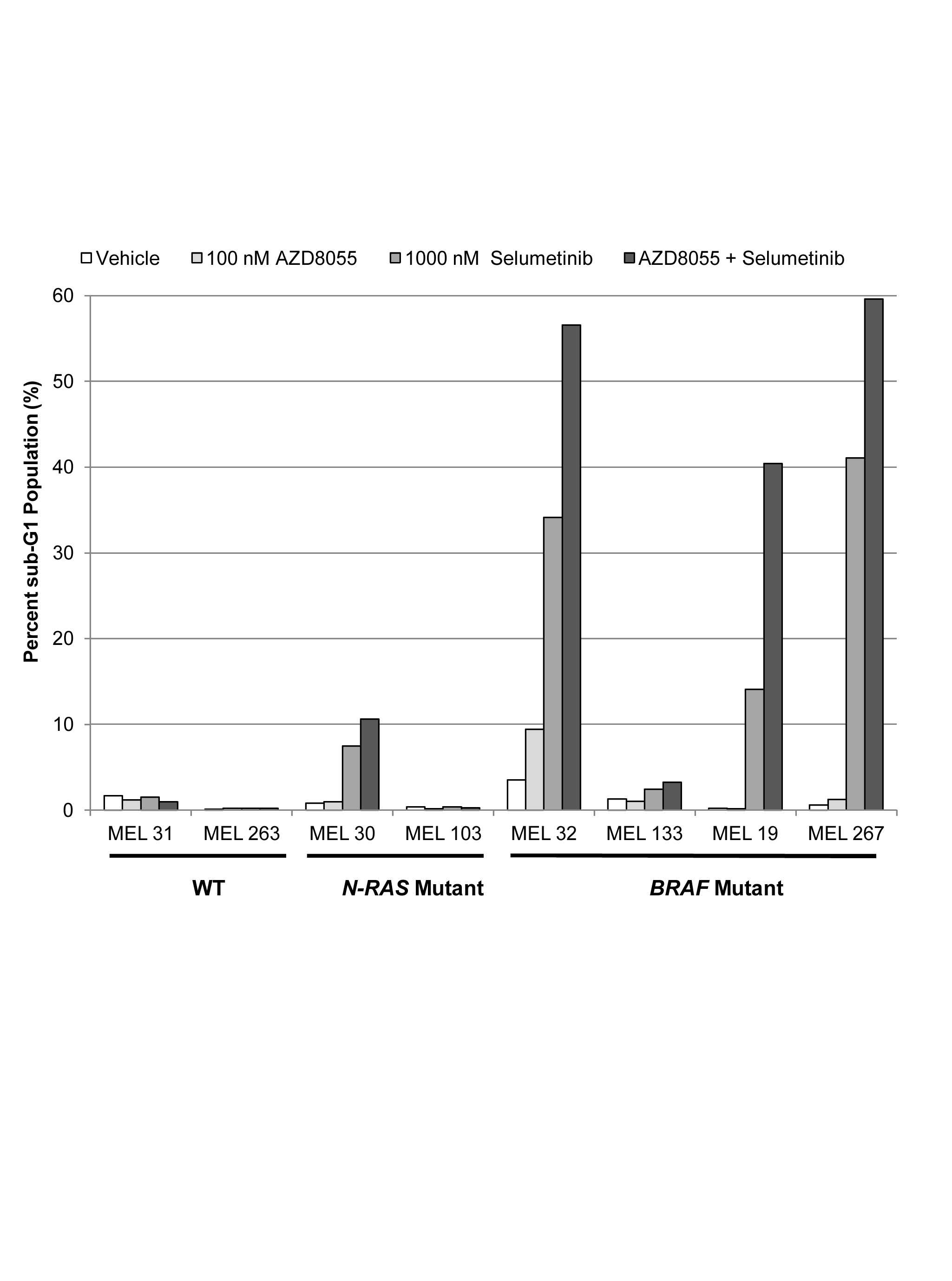

Supplement: Figure S9 — AZD8055/selumetinib induces apoptosis preferentially in BRAF mutant cutaneous melanomas. A panel of cutaneous melanoma cell lines of the indicated genotypes were treated with the indicated drugs for 72 hours (with the exception with MEL 267 which was treated for 48 hours) and then assayed by flow cytometry for DNA content in order to determine the percentage of cells with sub-G1 DNA, indicative of apoptosis. WT, defined as cells lacking BRAF, RAS, or any other known pathway mutations; N-RAS mutation, MEL 30 has a Q61K mutation and MEL 103 has a Q61R mutation in N-RAS; BRAF mutation, V600E. (TIF) [file pone.0040439.s009.tif]

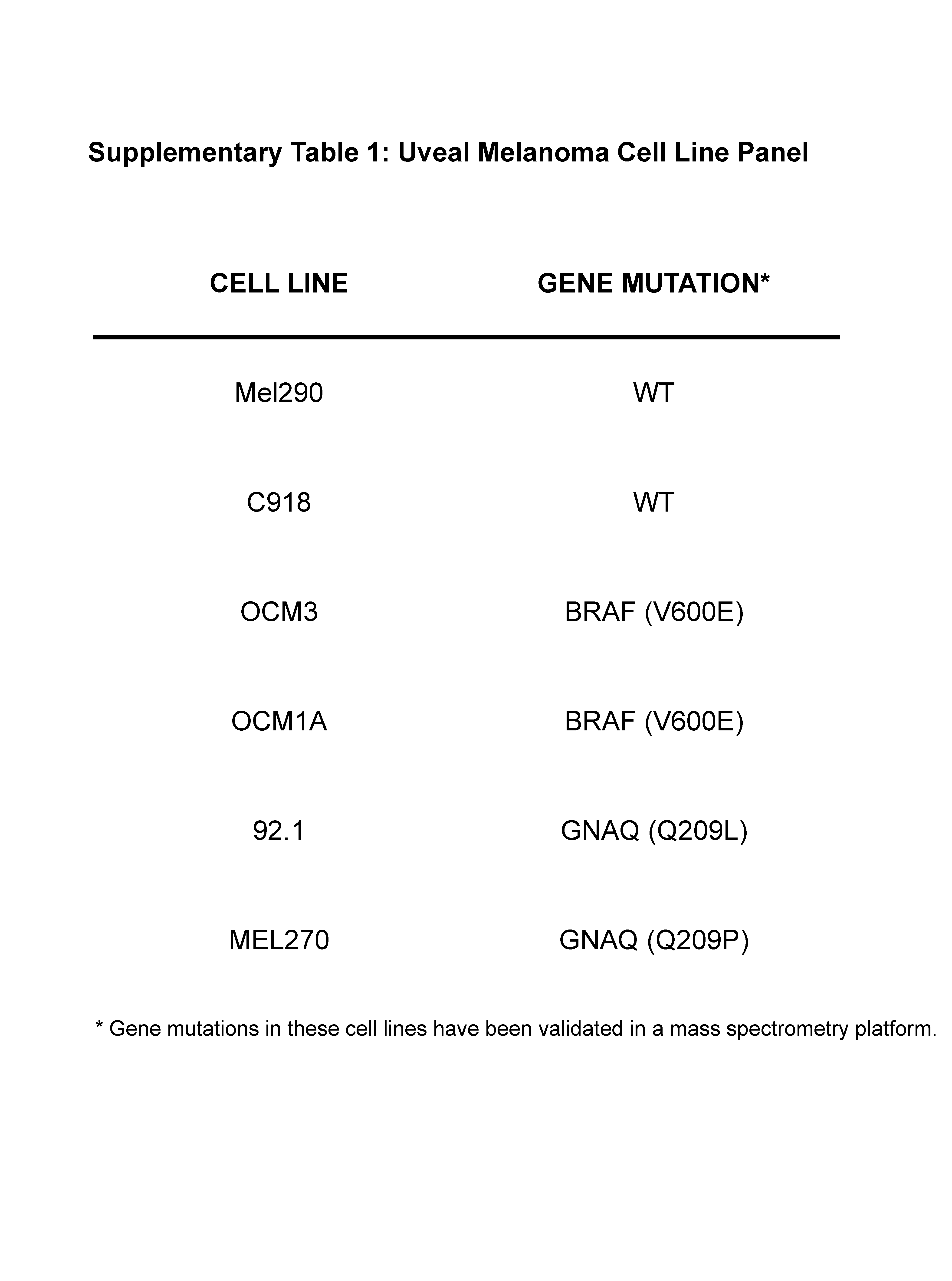

Supplement: Table S1 — List of the uveal melanoma cell lines utilized in this study. The gene mutations for each cell line listed were previously described/discovered by other groups. We also analyzed the cell lines on a mass spectrometry platform to assess for other hotspot gene mutations. Some of the relevant mutations tested include: AKT1 (E17K, R1114, Q1303, E1306*/K, E1308*/K, E1309*/K, E1322*, Q1338*, Q1367*, Q1367H, Q1378*, Q1379*, Q1406*, Q1429*, R1450*, S1465fs*3), PIK3CA (E542Q), ARAF, BRAF, EGFR, ERBB2, FGFR1-4, KIT, PDGFR A/B, KRAS, NRAS, HRAS, and MEK1. (TIF) [file pone.0040439.s010.tif]
